# Study of Burden in Polycystic Ovary Syndrome at Global, Regional, and National Levels from 1990 to 2019

**DOI:** 10.3390/healthcare11040562

**Published:** 2023-02-14

**Authors:** Yong Gao, Haobiao Liu, Lichun Qiao, Jiawei Liang, Haoyan Yao, Xue Lin, Yane Gao

**Affiliations:** 1 Department of Obstetrics & Gynecology, The Second Affiliated Hospital of Xi’an Jiaotong University, Xi’an 710004, China; 2 School of Public Health, Health Science Center, Xi’an Jiaotong University, Xi’an 710061, China; 3 Global Health Institute, Health Science Center, Xi’an Jiaotong University, Xi’an 712000, China

**Keywords:** polycystic ovary syndrome, GBD 2019, incidence, disability-adjusted life years, socio-demographic index

## Abstract

Increasing attention has recently been paid to the harm of polycystic ovary syndrome (PCOS) to women. However, due to the inconsistency of global clinical diagnostic standards and the differing allocation of medical resources among different regions, there is a lack of comprehensive estimation of the global incidence and disability-adjusted life years (DALYs) of PCOS. Thus, it is difficult to assess the disease burden. We extracted PCOS disease data from 1990 to 2019 from the Global Burden of Disease Study (GBD) 2019 and estimated the incidence, DALYs, and the corresponding age-standardized rates (ASRs) of PCOS, as well as the socio-demographic index (SDI) quintiles, to describe epidemiological trends at the global level, encompassing 21 regions and 204 countries and territories. Globally, the incidence and DALYs of PCOS have increased. Its ASR also shows an increasing trend. Among them, the high SDI quintile seems relatively stable, whereas other SDI quintiles are constantly rising over time. Our research has provided clues regarding the disease pattern and epidemic trend of PCOS and analyzed the possible causes of disease burden in some specific countries and territories, which may have some value in health resource allocation and health policy formulation and prevention strategies.

## 1. Introduction

Polycystic ovary syndrome (PCOS) is one of the most common endocrine and metabolic disorders in premenopausal women and is also the leading cause of anovulatory infertility. It is defined as the symptoms and signs of androgen excess and ovarian dysfunction after the exclusion of other diseases (including hyperprolactinemia, non-classical congenital adrenal hyperplasia, thyroid disease, hypogonadism, Cushing’s disease, or androgen-producing tumors) [1,2]. Although the exact cause remains unclear, it is generally accepted that lifestyle, environmental, and genetic factors play important roles in the occurrence and development of PCOS [3,4,5], which impairs women’s fertility and threatens their overall health [3]. Research [1] shows that the prevalence of PCOS under National Institutes of Health (NIH), Rotterdam and Androgen Excess and PCOS (AE-PCOS) Society criteria were 6.1, 19.9 and 15.3%, respectively. The harmful of PCOS cannot be ignored. Compelling evidence suggests that long-term complications of PCOS include endometrial cancer [6], hypertension [7], dyslipidemia [8], possible cardiovascular events [9], obesity [10], glucose intolerance, type 2 diabetes [11], metabolic syndrome [12], obstructive sleep apnea [13], non-alcoholic fatty liver disease [14], depression [15], anxiety [16], and pregnancy complications [17]. The harm of PCOS to female fertility and long-term health needs urgent attention without any dispute, and preventive and treatment measures should be taken immediately.

Doctors need to formulate individualized treatment plans according to the individual conditions of PCOS patients, along with close long-term follow-up management, posing great challenges to the treatment of PCOS [2]. As socioeconomic standards improve and access to medical resources increases, more attention is being paid to women’s reproductive health, and more people with PCOS are being found in the population. Nevertheless, there are not enough global and large-scale epidemiological studies on PCOS, especially regarding incidence and disability-adjusted life years (DALYs), leading to an unclear understanding of the burden of PCOS. Moreover, the insufficient number of reproductive endocrinologists prevents the PCOS population from receiving adequate professional medical care. Studies have shown differences in PCOS diagnosis and treatment patterns between obstetrician-gynecologists and reproductive endocrinologists [18,19].

To meet the needs of diagnosis and treatment of PCOS, government health management departments should allocate the corresponding medical resources according to the burden of PCOS and the disease situation in order to improve women’s quality of life and health. Although researchers have made significant efforts and achieved results, more large-scale, global burden studies of PCOS are still needed to guide the deployment of relevant specialists and the allocation of related healthcare resources. Therefore, we obtained relevant data concerning the burden of PCOS through the Global Burden of Disease Study (GBD) and analyzed the distribution and changing trend of incidence, DALYs, and corresponding age-standardized rate (ASR) of PCOS at the global, regional, and national levels from 1990 to 2019. At the same time, valuable information, such as the age distribution of PCOS, the relationship between the changing trend of disease burden and socio-demographic index (SDI), geographical region, and level of health care, is examined. The results show an increase in the incidence and DALYs of PCOS in individuals with PCOS worldwide. Although there are limitations, such as differences in diagnostic criteria for PCOS and the reliability of GBD data, this could be beneficial for health resource allocation and policy formulation regarding PCOS.

## 2. Materials and Methods

### 2.1. Summary

The GBD, led by the Institute for Health Metrics and Evaluation (IHME), provides the most comprehensive and up-to-date data assessment of the descriptive epidemiology of diseases in 21 regions and 204 countries and territories from 1990 to 2019, using all available data through global collaboration. This has made it one of the most important projects for understanding the global disease burden [20]. All data were calculated by direct query and downloaded from the GBD results tool. A detailed description of the methodology is available on the help page of the database and other publications [21]. The GBD collects health data from life records, registers, censuses, health surveys, population surveillance, scientific research, administrative reports, hospital discharge records, outpatient visits, and health insurance claims records, as well as many other sources. These are then input into algorithms to generate an estimate of the disease burden. In GBD studies, disease estimates were generated by age, year, and location using the Bayesian meta-regression tool DisMod-MR 2.1, ensuring the consistency of epidemiological parameters for the conditions studied [21].

### 2.2. Data Source

Data on the global burden of PCOS were obtained from published sources using the Global Health Data Exchange query tool. This study obtained data on the incidence, DALYs, and ASRs of PCOS at the global, regional, national, and SDI quintiles from 1990 to 2019 from the GBD 2019. GBD divides the SDI of 21 regions and 204 countries and territories into five parts (high, high-middle, middle, low-middle, and low) according to total fertility rate, per capita income, and average years of education [22]. SDI ranges from 0 to 1, and a larger value indicates a higher level of socioeconomic development. Additionally, GBD regions are not actual geopolitical units. Rather, they are groupings of countries created for analysis. The international classification of disease (ICD) code 10 (ICD-10 codes, E28.2) and ICD-9 code (E28.2) were used to identify cases of PCOS.

### 2.3. Statistical Analysis

ASR and estimated annual percentage change (EAPC) were calculated to quantify and compare incidence and DALYs trends of PCOS. Standardization can eliminate the effects of the population’s age composition and ensure the comparability of research indicators. The following formula calculates ASR (per 100,000 population):

(1) $$\mathrm{ASR}=\frac{\sum_{i=1}^{A}aiwi}{\sum_{i=1}^{A}wi}\times 100,000$$

where *ai* and *wi* represent specific age ratios and the number of people (or body weight) in the same age subgroup of the selected reference standard population (where *i* represents the *i*-th age group), respectively, and *A* is the number of age groups. The EAPC is used to quantify the trend in ASR within the designation and is calculated using a generalized linear model with a Gaussian distribution. The regression line is fitted to the natural logarithmic rate, Y = α + βx + ε, where x = year and y = ln(ASR), and the formula for calculating EAPC is:

(2) EAPC=100×(exp(β)−1)

(The 95% confidence interval of EAPC was obtained from the linear model).

Positive EAPC and lower bound of its 95% CI indicate that ASR had an upward trend, and negative EAPC and upper bound of its 95% CI indicate that ASR had a downward trend. Otherwise, the ASR is deemed to be stable over time.

In addition, we explored the relationship between EAPC and ASR (1990) and the human development index (HDI) (2019) at the national level, respectively, to find the potentially influential factors of EAPC. HDI was proposed by WHO in 1990 and calculated based on certain methods from three basic variables: life expectancy, education level, and quality of life.

All statistical analyses and graphing were performed using EXCEL 2019 (Microsoft Corporation) and R software (version 4.1.0). *p* < 0.05 was considered statistically significant.

## 3. Results

### 3.1. The Burden of PCOS at the Global Level

**Figure 1:**
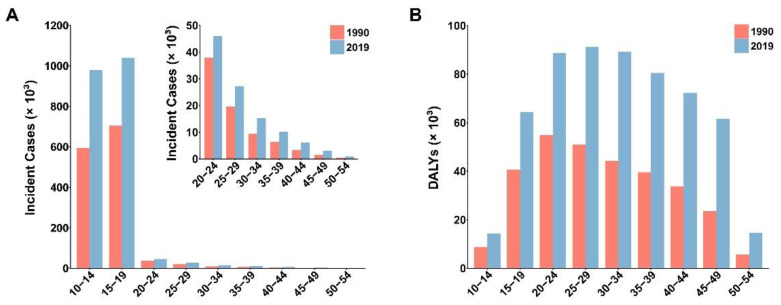
The incident cases (**A**) and DALYs (**B**) of PCOS at different age groups in 1990 and 2019. DALYs, disability-adjusted life years; PCOS, polycystic ovary syndrome.

Globally, the incident cases of PCOS grew by 54% from 1,377,924.58 [95% uncertainty interval (UI): 941,751.00–1,816,993.69] in 1990 to 2,125,511.75 (95% UI: 1,489,953.81–2,803,300.68) in 2019 (Table 1). As shown in Figure 1A, the number of PCOS cases decreased gradually by age, with the highest incident cases in the 15–19 group, followed by the 10–14 age group. In addition, we found that the number of incident cases for each age group in 2019 was higher than those in 1990 to varying degrees. The global age-standardized incidence rate (ASIR) showed an increasing trend (EAPC: 0.85, 95% CI: 0.83–0.87) from 46.14 (95% UI: 31.62–61.04) per 100,000 to 59.76 (95% UI: 41.71–78.87) per 100,000. Furthermore, there were 576,822.00 (95% UI: 246,221.54–1,161,158.56) DALYs in 2019, an increase of 91% (95% UI: 0.83–0.99) relative to 301,987.02 (95% UI: 127,370.28–606,719.05) in 1990 (Table 2). Figure 1B demonstrates that for all age groups, DALYs were greater in 2019 than they were in 1990. The 25–29 age group had the greatest DALYs in 2019, followed by the 30–34 age group. PCOS-related DALYs were higher in women of childbearing potential aged 20–40 years, but lower in adolescent girls aged 10–14 years and perimenopausal women aged 50–54 years. The age-standardized disability-adjusted life years rate (ASDR) showed a smaller increase from 11.30 (95% UI: 4.78–22.67) per 100,000 in 1990 to 14.68 (95% UI: 6.28–29.45) per 100,000 in 2019. The overall trend in ASDR was upward (EAPC: 0.83, 95% CI: 0.78–0.87).

### 3.2. The Burden of PCOS at the Regional Level

**Figure 2:**
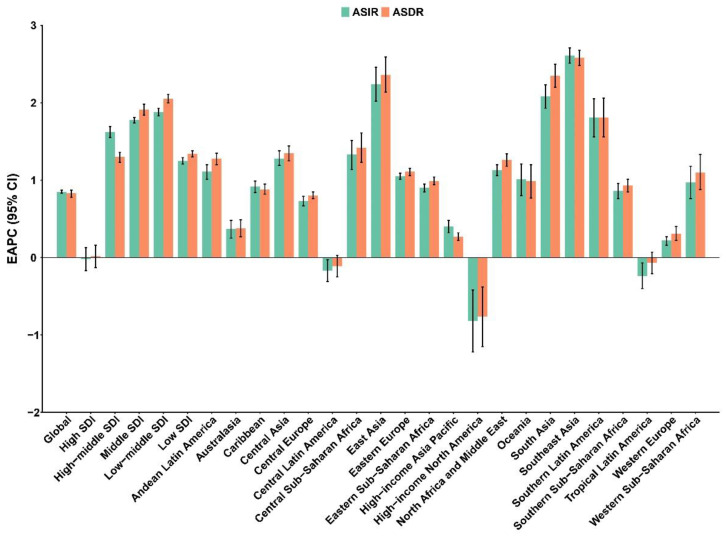
The EAPC for ASIR and ASDR of PCOS at the regional level. EAPC, estimated annual percentage change; ASIR, age-standardized incidence rate; ASDR, age-standardized disability-adjusted life years rate; PCOS, polycystic ovary syndrome.

Regionally, incident cases increased in most regions except Central Europe, Eastern Europe, high-income Asia Pacific, and Western Europe. Compared to 1990, incident cases increased 2.87−fold (95% UI: 2.54–3.30) and 2.62-fold (95% UI: 2.44–2.82) in central Sub-Saharan Africa and western Sub-Saharan Africa in 2019, respectively, while they decreased in Central Europe and high-income Asia Pacific and did not change significantly in Eastern Europe and Western Europe. In high-income North America (EAPC: −0.82, 95% CI: −1.22–−0.42), tropic Latin America (EAPC: −0.24, 95% CI: −0.40–−0.07), and central Latin America (EAPC: −0.17, 95% CI: −0.31–−0.03), ASIR showed a decreasing trend over time. In contrast, all other regions showed an increasing trend in ASIR, with the largest increase in Southeast Asia (EAPC: 2.61, 95% CI: 2.51–2.71), followed by East Asia (EAPC: 2.24, 95% CI: 2.02–2.46) (Table 1, Figure 2). From 1990 to 2019, DALYs increased in all regions except Central Europe and high-income Asia Pacific. Central Sub-Saharan Africa had the largest increase among the 21 regions, with a 2.96-fold (95% UI: 2.51–3.49) increase, and western Sub-Saharan Africa had a 2.90-fold (95% UI: 2.68–3.14) increase. Among the changes in ASDR, the most pronounced increase was in Southeast Asia (EAPC: 2.58, 95% CI: 2.48–2.68). In addition, there was a downward trend in ASDR in high-income North America (EAPC: −0.76, 95% CI: −1.15–−0.38) and a relatively stable trend in central Latin America (EAPC: −0.11, 95% CI: −0.25–0.03) and tropical Latin America (EAPC: −0.07, 95% CI: −0.21–0.07) (Table 2, Figure 2).

### 3.3. The Burden of PCOS at the National Level

Nationally, the top five countries with high incident cases in 2019 were India (332,699.82, 95% UI: 225,124.51–441,702.27), China (223,654.29, 95% UI: 152,542.05–298,625.60), the United States of America (157,225.76, 95% UI: 125,037.43–193,575.59), Indonesia (135,161.55, 95% UI: 92,064.01–180,625.53), and Japan (89,573.34, 95% UI: 60,126.15–124,407.16). The top five countries with low incident cases were Tokelau, Niue, Tuvalu, Nauru, and Palau. Moreover, the top five countries with high ASIR in 2019 were Italy (314.44/100,000), followed by Japan (264.33/100,000), New Zealand (226.24/100,000), Australia (192.80/100,000), and Malaysia (162.11/100,000), while the top five countries with low ASIR were southeast European countries, including Bosnia and Herzegovina (7.36/100,000), Albania (7.39/100,000), North Macedonia (7.54/100,000), Serbia (7.56/100,000), and Romania (7.98/100,000). Over the 30 years, the United States of America, Italy, Mexico, Brazil, and New Zealand exhibited decreasing trends in ASIR. Zimbabwe, Central African Republic, and Austria were relatively stable, while ASIR in the remaining 196 countries increased to varying degrees (see Supplementary Materials, Table S1).

The countries ranked among the top five in 2019 for DALYs were China (90,036.27, 95% UI: 38,360.94–181,836.22), India (79,398.96, 95% UI: 33,651.92–161,428.50), the United States of America (42,738.54, 95% UI: 19,395.20–82,347.03), Indonesia (33,613.48, 95% UI: 14,439.19–68,811.04), and Japan (28,279.15, 95% UI: 11,811.56–59,132.75). In addition, the top five countries with high ASDR were Italy (69.70/100,000), Japan (54.81/100,000), New Zealand (47.17/100,000), Australia (39.25/100,000), and Malaysia (36.23/100,000). From 1990 to 2019, the United States of America, Mexico, Italy, and New Zealand showed declining trends in ASDR. Zimbabwe, Brazil, Austria, and Central African Republic were relatively stable, and the remaining 196 countries showed an upward trend in ASDR (see Supplementary Materials, Table S2).

### 3.4. The Burden of PCOS at the SDI-Quintile Level

**Figure 3:**
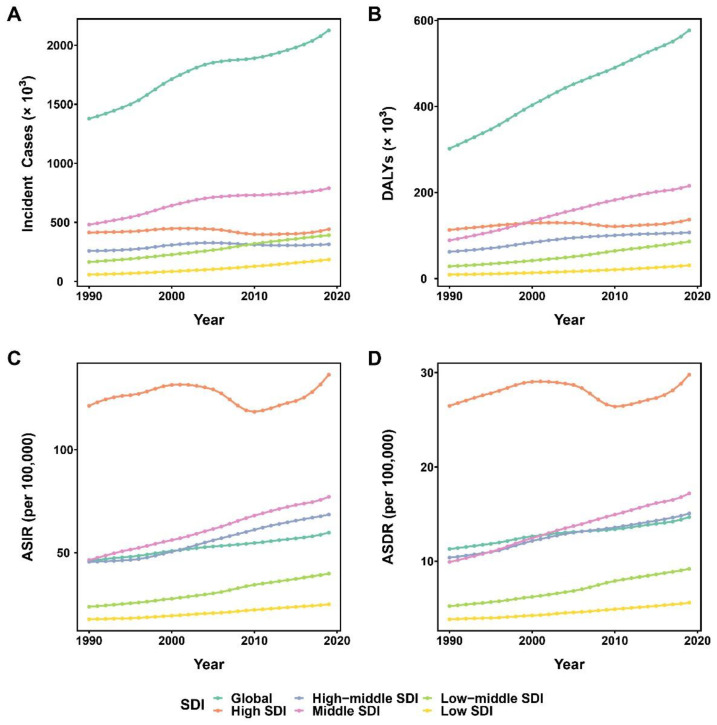
The changes in cases and ASR of PCOS in global and different SDI quintiles from 1990 to 2019. (**A**) Changes in incident cases of PCOS; (**B**) Changes in DALYs of PCOS; (**C**) Changes in ASIR of PCOS; (**D**) Changes in ASDR of PCOS. ASR, age-standardized rates; PCOS, polycystic ovary syndrome; SDI, socio-demographic index; DALYs, disability-adjusted life years; ASIR, age-standardized incidence rate; ASDR, age-standardized disability-adjusted life years rate.

Globally, the incident cases and DALYs of all SDI quintiles increased in 2019 compared to 1990, but incident cases of the high SDI quintile did not increase significantly. The incident cases in the high and high-middle SDI quintiles and the DALYs in the high SDI quintile did not increase unidirectionally and showed some fluctuation over this period. In particular, incident cases in the middle SDI quintile remained highest from 1990 to 2019. DALYs in the high SDI quintile were highest before 1999, while those in the middle SDI quintile were highest from 1999 to 2019. Throughout the period, the incident cases and DALYs in the high SDI quintile showed small fluctuations and remained at a certain level. In addition, the incident cases and DALYs in the low SDI quintile remained consistently at the lowest level (Table 1, Figure 3A,B).

**Figure 4:**
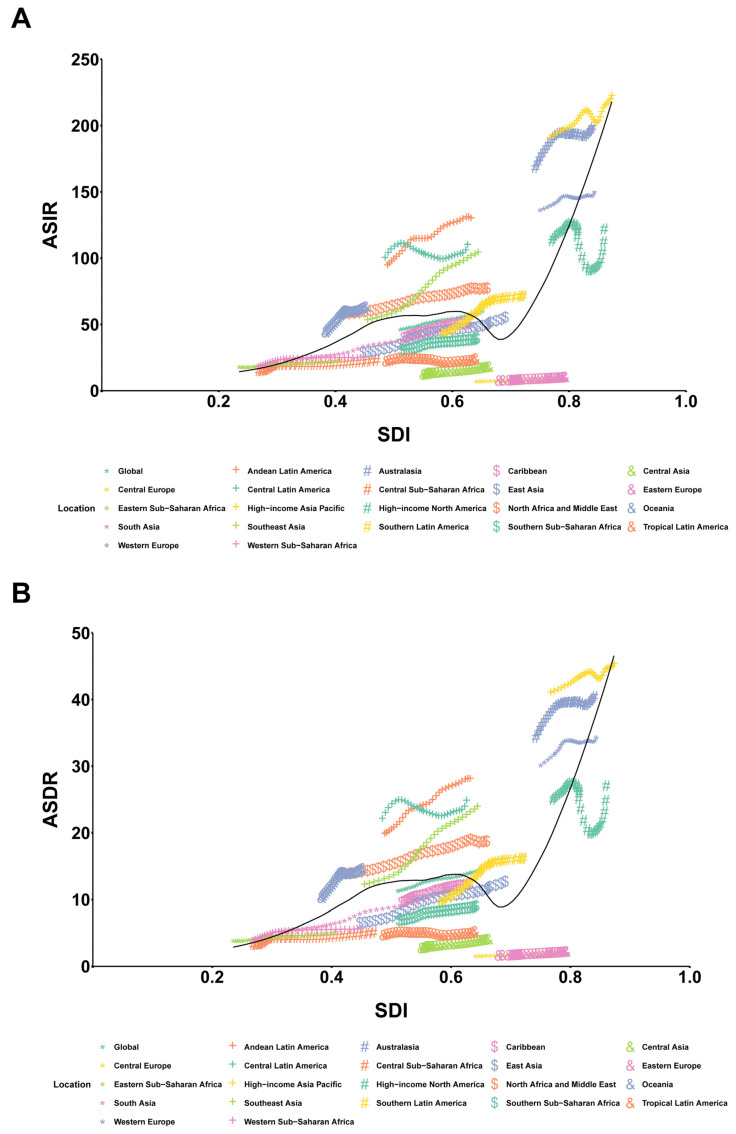

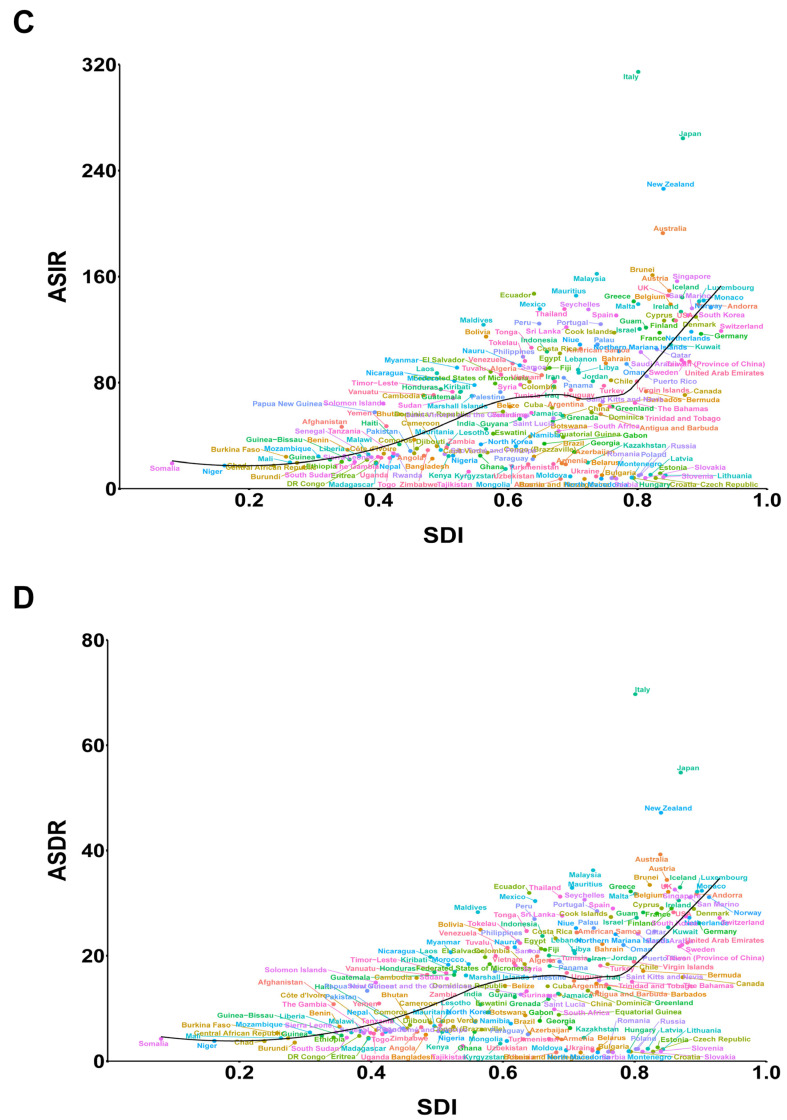
Correlation between SDI and ASR of PCOS at regional and national levels. (**A**) Correlation between SDI and ASIR (per 100,000) at the regional level from 1990 to 2019. (**B**) Correlation between SDI and ASDR (per 100,000) at the regional level from 1990 to 2019. (**C**) Correlation between SDI and ASIR at the national level in 2019. (**D**) Correlation between SDI and ASIR at the national level in 2019. (**A**,**B**) Colored lines show global and region values for age-standardized incidence rates. Each point in a line represents 1 year starting in 1990 and ending in 2019. The black line represents the average expected relationship between SDI and ASR for PCOS from all regions from 1990 to 2019. (**C**,**D**) Each colored dot above represents a country. The black line represents the average expected relationship between SDI and ASR. SDI, socio-demographic index; ASR, age-standardized rates; PCOS, polycystic ovary syndrome; ASIR, age-standardized incidence rate; ASDR, age-standardized disability-adjusted life years rate.

From 1990 to 2019, the ASIR and ASDR of PCOS increased in all SDI quintiles but fluctuated more in the high SDI quintile. The high quintile remained above the global level, the middle and high-middle quintiles were close to it, and the low and low-middle quintiles remained below it. Over the 30 years, the global ASIR showed an upward trend (EAPC: 0.85, 95% CI: 0.83–0.87), with the greatest increase in the low-middle SDI quintile (EAPC: 1.88, 95% CI: 1.83–1.93) and relative stability in the high SDI quintile (EAPC: −0.02, 95% CI: −0.17–0.13) (Table 1, Figure 3C). Moreover, the global ASDR exhibited an upward trend (EAPC: 0.83, 95% CI: 0.78–0.87), with the largest increase occurring in the low-middle SDI quintile (EAPC: 2.05, 95% CI: 2.00–2.11) and the high SDI quintile remaining relatively stable (EAPC: 0.02, 95% CI: −0.13–0.16) (Table 2, Figure 3D). According to GBD 2019 data, the relationship curves between SDI and ASR were fitted for different regions and countries/territories, and a positive nonlinear correlation was found between SDI and ASR (ASIR and ASDR). Regionally, with an increase in SDI, ASR initially increases, then decreases, and finally increases drastically. Nationally, ASR has a general upward tendency as SDI rises (Figure 4).

### 3.5. The Influential Factors for EAPC

**Figure 5:**
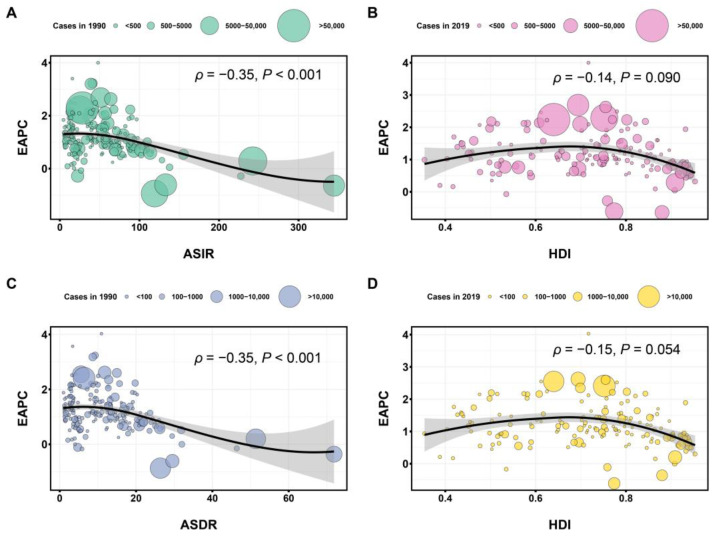
The correlation between EAPC and ASR (per 100,000) in 1990, as well as EAPC and HDI of PCOS in 2019. (**A**) Correlation between EAPC and ASIR (per 100,000) in 1990. (**B**) Correlation between EAPC and HDI in incidence in 2019. (**C**) Correlation between EAPC and ASDR (per 100,000) in 1990. (**D**) Correlation between EAPC and HDI in DALYs in 2019. Each circle represents a country or region, and the circle size positively correlates with the number of PCOS cases in that country or region. EAPC, estimated annual percentage change; ASR, age-standardized rates; HDI, human development index; PCOS, polycystic ovary syndrome; ASIR, age-standardized incidence rate; ASDR, age-standardized disability-adjusted life years rate.

The ASR in 1990 reflects the baseline level of PCOS, and the HDI in 2019 can be used as a surrogate for the level and availability of health care in each country and territory. When ASIR or ASDR increases to a high level, EAPC would include 0, the lower bound of its 95% CI is <0, and the upper bound of its 95% CI is >0, which means that ASIR and ASDR are deemed to be stable over time. As shown in Figure 5, negative correlations were detected between EAPC and ASIR (ρ = −0.35, *p* < 0.001) or ASDR (ρ = −0.35, *p* < 0.001) in 1990. However, there were no correlations between EAPC and HDI in incidence (ρ = −0.14, *p* = 0.090) and DALYs (ρ = −0.15, *p* = 0.054) in 2019.

## 4. Discussion

PCOS is a complex, chronic, and under-recognized disease. Delayed diagnosis and lack of information are very common issues for women with PCOS. According to a study by Gibson-Helm et al. [23], only 35.2% and 15.6% of PCOS patients were satisfied with their diagnosis experience and the information obtained during their doctor visit, respectively. Seeing ≥5 health professionals and longer time to diagnosis were negatively associated with diagnosis satisfaction. PCOS causes a significant impact on women’s quality of life, including difficulties in losing weight, irregular menstrual cycles, infertility, and hirsutism, which should attract the attention of academics and government health administrators.

### 4.1. Global Burden of PCOS and Its Possible Causes

In 2021, a GBD-based study by Liu et al. [24] showed that slight increases in ASIR of PCOS and DALYs were evidenced among women of reproductive age (15–49 years) from 2007 to 2017, without showing the trend of continuous changes in the burden during this period, in addition to the discussion focusing on social factors in some countries. The study [24] showed that the global incident cases and DALYs of PCOS were 15,545,089 (95% UI: 1,190,519–2,081,418) and 426,361 (95% UI: 189,151–820,948), respectively, in 2017. Unusually, in this study, we included a longer period of changes, while estimating the disease burden and analyzing its temporal trends at the global, regional, and national levels based on the GBD 2019. In 2019, there were approximately 2.12 million incident cases and 0.58 million DALYs worldwide, both significantly higher than that in 1990 and 2017. As disease data are updated, we would like to observe whether the burden of PCOS has altered markedly. In addition, it is noted that GBD2019 also includes people aged 10–14 and 50–54, with a high incidence in the 10–14 group and DALYs in the 50–54 group. Our study described the burden trends over the past 30 years from 1990 to 2019, with the longer time- and age-span better reflecting the characteristics of PCOS burden. We also attempted to point out the characteristics and causes of PCOS burden from SDI quintiles, to broadly predict trends of PCOS burden in some countries and regions. Over the past 30 years, we have observed a significant increase in ASIR globally, and the burden of PCOS differs across SDI quintiles. The increase in incident cases and DALYs was most pronounced in the middle SDI quintile, but ASIR and ASDR presented a small increase, which may be related to the significant changes in population structure. Patients with PCOS in the low SES may encounter obstacles when obtaining specialist health care, affecting the diagnosis and treatment of PCOS to some extent. A large-scale retrospective study [25] and a systematic study [26] showed that socioeconomic status (SES) was associated with access to health care services, with people of higher SES being more likely to have access to specialist health care. Moreover, a cross-sectional study also conducted that income and education were the top two factors influencing access to specialist health care [27]. Treatment plans for PCOS must be individualized, and long-term clinical management is essential [28]. For the middle SDI quintile, difficulties in persisting with follow-up and long-term clinical management after being diagnosed may account for the particularly high DALYs in PCOS. Additionally, low SES is associated with common PCOS symptoms or comorbidities, such as obesity and insulin resistance [29,30], which explains the high incident cases in the middle SDI quintile. Furthermore, the low SDI quintile ASIR has remained the lowest and grown at the lowest rate among all SDI quintiles. On the one hand, these populations still have difficulty obtaining a PCOS diagnosis. On the other hand, a series of social factors, such as relatively less environmental pollution and less stress, may account for their low ASIR. In terms of overall trends, ASIR and ASDR in the high SDI quintile appear to fluctuate around a baseline, indicating that the burden of PCOS in the high SDI quintile may tend to be stable.

### 4.2. Regional Burden of PCOS and Its Possible Causes

Regionally, South Asia had the most incident cases in 2019, as India had the highest incident cases of PCOS in the world. East Asia had the highest DALYs among the 21 regions, mainly because China ranks first among all 204 countries and territories, contributing the majority of DALYs to this region. Meanwhile, high-income Asia Pacific has the highest ASIR and ASDR, partly attributed to the highly developed economy and availability of medical resources in this region. On the other hand, the current diagnostic criteria for PCOS were established without considering different ethnicities. East Asians have a lower threshold for hirsutism compared to Caucasians. It is observed that “albeit given the differences in diagnostic criteria for polycystic ovary morphology (PCOM) on ultrasound, the proportion of East Asian women with PCOS having PCOM on ultrasound appeared to be higher (92.9% vs. 69.9%) than Caucasian women with PCOS” [31], which may contribute to the high incidence of PCOS in East Asians. Additionally, all countries in the high-income Asia Pacific belong to the high SDI quintile, which is consistent with finding that the high SDI quintile has the highest ASIR and ASDR. Although Central Europe and Eastern Europe fall within the high SDI quintile, they showed lower ASIR and ASDR. This suggests that we should consider the correlation between genes, lifestyle, dietary habits, nutritional intake, socio-cultural and geographical environment, and low ASIR in these regions when seeking reasons for the low disease burden.

### 4.3. National Burden of PCOS and Its Possible Causes

Nationally, India, China, the United States of America, Indonesia, and Japan were the top five countries with high incident cases and DALYs of PCOS in 2019 and the previous several years, all of which have large population bases inextricably linked to their high incident cases and DALYs. India is a middle SDI country, and its burden trend is consistent with the overall trends of the middle SDI quintile. Nevertheless, the United States and Japan belong to high SDI countries, and China and Indonesia are middle SDI countries, all of whose trends differ from their SDI quintiles. The United States has the third-largest population in the world. Although it has the most advanced medical standard in the world, its health insurance system cannot benefit the majority of people. High medical costs and excessive administrative burdens on healthcare professionals lead to the inefficient operation of the healthcare system. Moreover, the strong correlation between income and health results in no benefit from high-quality health care for low- and middle-income Americans [32]. Instead, bankruptcy due to health care costs or inability to access health care for lack of health insurance is common [33]. With a fast-paced lifestyle, the Japanese are often under stress caused by long working hours, heavy workloads, and interpersonal conflicts with supervisors or managers, which may lead to a higher incidence of mental disorders in the population [34]. Especially for women, the odds of persistence of any common mental disorder are higher, and the treatment rate for patients with moderate mental disorders has recently improved in Japan [35]. Sodium valproate, a commonly used psychotherapeutic drug, has been closely associated with PCOS [36,37,38,39]. Although some current recommendations for the cautious use of valproate exist, there are no strong binding regulations prohibiting the use of valproate [40], which may be related to the current high incidence of PCOS in Japan. Statistics from the Japanese Ministry of Health, Labor, and Social Security showed that nearly 128 million people were struggling with their weight and an estimated 23 million were obese due to the disappearance of the traditional rice and fish-centered diet, the prevalence of cheap fast food and increasingly sedentary lifestyles [41], which are also closely related to PCOS. In addition, the new diagnostic criteria for PCOS adopted in Japan may also contribute to its high incident cases and DALYs [42].

As a developing country with uneven regional development, China has a higher percentage of medical insurance reimbursement for high-income people than for low-income groups, which leads to poorer health conditions for the latter [43]. However, since PCOS is not seriously life-threatening in the short term, a larger proportion of the low-income population may refuse standardized and reasonable specialist follow-up and treatment after being diagnosed. Moreover, China and Indonesia are rapidly developing, and their huge investment in medical and health services has substantially boosted the rate of PCOS screening. We have noted some of the initiatives proposed by the Chinese government in the “Outline of Healthy China 2030” [44] and “Outline of Chinese Women and Children’s Development, 2021–2030” [45], such as implementing a program to promote physical activity among youth and ensure that students have at least one hour of activity time per day; achieving a balanced allocation of high-quality basic medical and health resources; improving the health of women and children; increasing the screening rate and early diagnosis and treatment of common diseases among women and so on. These would be beneficial in the prevention and treatment of PCOS. The relevant policies have certain reference significance for those countries with a high disease burden of PCOS.

Italy, Japan, New Zealand, Australia, and Malaysia occupied the top five ASIR and ASDR for PCOS. Among them, all except Malaysia were high SDI countries, and the higher disease burden they exhibit is consistent with the overall trend in the high SDI quintile. Malaysia had a large increase in ASIR from 1990 to 2019. A cross-sectional study [46] showed that overweight and obesity were particularly prominent among adolescents and young people aged 16–35 years in Malaysia, with an overweight rate of 12.8% and obesity rate of 7.9% for those aged 16–20 years and an overweight rate of 7.9% and obesity rate of 20.9% for those aged 31–35 years. Numerous studies have concluded a strong association between insulin resistance due to being overweight and obesity and PCOS [47,48,49]. Over the past 30 years, there have been lifestyle changes, especially among middle-class women who face interruptions in diet and sleep habits, new pressures on women from competition for education and work, consumption, and the reconciliation of family and work responsibilities. A study by Indian scholars showed that these burdens were related to the increased incidence of PCOS [50]. This also indicates that the number of patients with PCOS requiring specialist health care will increase in the coming period as society develops and progresses. At the same time, countries and territories experiencing rapid growth in SDI should also adopt related policies to accommodate the increasing healthcare needs of the increasing number of PCOS patients.

According to research, the number of women aged 10–19 years old accounts for almost the majority of incident cases in the total female population, which provides some pointers for preventing PCOS. This population is generally students, making it possible to interfere in their lifestyle through a series of measures, such as diet, nutrition, sleep, physical activity, and vitamin and micronutrient supplementation [51,52,53,54], which can be of great benefit to women’s health. On the other hand, the applicability of the previous diagnostic criteria for PCOS to this population needs to be reconsidered and perhaps a new diagnostic protocol for this population should be formulated.

### 4.4. Both Doctors and Patients Need to Make Efforts to Deal with PCOS

PCOS should be diagnosed and treated by specialized, trained gynecologic endocrinologists, but most developing countries do not have enough gynecologic endocrinologists to meet their needs. In the past decade, the need to improve the treatment and follow-up of patients has become increasingly apparent. Faced with this situation, some patients have turned to obstetricians and gynecologists for help, but they cannot access adequate expertise, diagnosis, and treatment services. Moreover, research on PCOS is increasing, awareness is growing, and doctors must receive timely training to guide clinical practice according to the latest guidelines. An online survey [18] of gynecologists and reproductive endocrinologists’ knowledge of PCOS diagnostic criteria and management in North America showed that a large proportion of respondents were unaware of the currently recommended diagnostic criteria. Only 68.3% of reproductive endocrinologists and 41.2% of gynecologists applied the Rotterdam criteria; and fewer gynecologists were aware of PCOS-related depression, anxiety, and decreased quality of life; and only 41.6% of gynecologists recommending lifestyle changes for PCOS patients [18]. According to an online cross-sectional survey conducted in China, less than one third of Chinese obstetricians and gynecologists could accurately diagnose PCOS using diagnostic criteria [55]. For countries and regions with insufficient numbers of reproductive endocrinologists, there is an urgent need to enhance the continuing education of obstetricians and gynecologists on the diagnosis and treatment of PCOS and the staff training of reproductive endocrinologists to improve the quality of healthcare services for PCOS. The available raw data may be skewed to some extent for countries and regions lacking well-trained reproductive endocrinologists.

There remains no universal treatment for PCOS, and treatment programs should be formulated according to the different situations of individuals. However, whatever the situation is, lifestyle intervention is the most basic and necessary measure for patients. Due to the close correlation between obesity and PCOS, reasonable physical exercise and healthy eating habits are necessary for patients to prevent or treat obesity. Sometimes, it is not enough to rely on the doctor’s support and the patient’s perseverance. The doctor can set up a mutual support group to increase the compliance of PCOS patients, so that they can maintain a good lifestyle and achieve the purpose of long-term lifestyle intervention. Studies [56,57] showed that lifestyle interventions could significantly improve free androgen index, body weight, BMI, depression, and health-related quality of life in women. In most studies, weight loss significantly improves the PCOS profile regardless of diet [58]. In the long run, lifestyle intervention is an effective treatment for PCOS. In terms of drug treatment, combined hormonal contraceptives (CHC) are often the first-line treatment for many physicians. A systematic review [59] found that PCOS was a risk factor for VTE after adjustment analyses for obesity and hormonal therapy (OR 1.89; 95% CI 1.60–2.24). However, another systematic study [60] showed that women with PCOS had a two-fold increased risk of VTE when using combined oral contraceptives (COC) in combination with second or third generation progestins (RR = 2.14; 95 95% CI, 1.41–3.24), and PCOS women with hyperandrogenism showed a higher risk (HR = 2.49; 95% CI, 1.35–4.59). Although the included patients have different PCOS phenotypes and the confounding factors are not controlled, there is no doubt that the use of CHC increases the risk of VTE, and thus the occurrence of VTE during treatment significantly increases the disease burden of PCOS. It cannot be ignored that PCOS patients are often accompanied by high BMI, which is one of the high-risk factors for VTE. If there is no correct exclusion of contraindications and a lack of standardized monitoring and follow-up during CHC use, thrombosis would become a life-threatening problem for patients, not PCOS. Therefore, it is very important to dynamically evaluate the risk of thrombosis and cardiovascular events in patients during the treatment.

### 4.5. Limitations and Challenges

Simultaneously, we must be aware of the study’s limitations. To date, the GBD is the most standard and authoritative system for estimating the global disease burden, with uniform and standardized data analysis and model building in line with the latest statistical analysis techniques. The limitations of GBD in disease burden analysis are mainly in the reliability of original data and statistical methods [21]. Due to the limited availability of incidence and DALYs data, burden estimates for most countries are based on models rather than actual values. Although GBD ensures that the model meets the real situation by collecting as much data as possible, the existing data sources are still limited, and the data quality is uneven at various national and regional levels. In some countries, health data reporting is relatively lagging and some data may not be available for estimation and modeling by the GBD [61], which may affect the accuracy of disease burden prediction. Unfortunately, the etiological data of PCOS are not included in GBD. Although some countries have shown high burden of PCOS, we have also tried to find relevant reasons to explain them, but we have to admit that the diagnostic ability, SES, medical insurance system, and education level all affect the disease burden data in the GBD to some extent. Therefore, the reason we provide is, to a certain extent, only speculation, which may be of some value to the intervention of PCOS in these countries and regions. For the burden of PCOS in a specific country or region, researchers still need to conduct relevant research and increase their efforts regarding this issue. Most importantly, the diagnostic criteria for PCOS are not unified worldwide, and the three current diagnostic criteria were proposed by three organizations, the National Institutes of Health (1990) and Rotterdam PCOS Consensus Workshop Group (2004), and Androgen Excess and PCOS Society (2006). Therefore, different diagnostic criteria might correspond to the same ICD code (E28.2) during the data statistics and analysis. Of these, the Rotterdam criteria currently represent the most accepted diagnostic approach, encompassing a broader spectrum of evidence of PCOS, including oligomenorrhea/amenorrhea, clinical/biochemical androgenic, and PCOM. However, using the Rotterdam criteria as a diagnostic method increased the prevalence of PCOS compared with the other two criteria, since clinical/biochemical androgen excess is not necessary for diagnosis. In addition, guidelines and consensus for PCOS management are constantly changing. Upgrades in ultrasound equipment have allowed physicians to see more and more follicles, and international PCOS guidelines [62] now recommend increasing the limit of polycystic ovaries to >20 follicles. A study has shown that combining anti-Mullerian hormone and clinical symptoms exhibits a satisfactory diagnostic potential for PCOS [63]. It is foreseeable that various relevant research advances will constantly change the diagnostic criteria as PCOS continues to be better studied and understood.

PCOS was first described by Stein and Leventhal in 1935 [64]. It was not until 1990 that the first accepted diagnostic criteria for PCOS were proposed. With the emphasis on women’s health, there has been an increasing number of relevant studies on the etiology, pathophysiology, molecular biology, genetics, and sociology of PCOS. The present study found that Italy, Japan, New Zealand, and Australia exhibit an extremely high disease burdens of PCOS, while it remains low in Western and Central Europe. In future research on PCOS, we will focus on these two populations and apply various research methods to conduct relevant research in order to obtain important findings.

## 5. Conclusions

From 1990 to 2019, the disease burden of PCOS increased worldwide. Its incident cases, DALYs, and corresponding ASRs appeared to fluctuate around a baseline, as observed in the high SDI quintile. It is not difficult to see that the overall trend shows an increase when comparing the data between 1990 and 2019. In addition, we found that the ASDR of PCOS increased at a slower rate in countries and territories with a higher HDI. We also analyzed the causes of PCOS disease burden in some countries and territories, which may have certain reference value for local health decision-making. Finally, we found that Italy, Japan, New Zealand, and Australia showed extremely high PCOS disease burdens, while the PCOS disease burden in Central and Eastern Europe was extremely low, making these regions suitable research subjects for future studies on PCOS.

## Figures and Tables

**Table 1:** The incident cases and ASIR of PCOS at the regional level and its temporal trends from 1990 to 2019.

Characteristics	1990		2019		1990–2019	
	Incident Cases No. (95% UI)	ASIR per 100,000 No. (95% UI)	Incident Cases No. (95% UI)	ASIR per 100,000 No. (95% UI)	Change in Incident Cases No. (95% UI)	EAPC No. (95% CI)
Global	1,377,924.58 (941,751.00–1,816,993.69)	46.14 (31.62–61.04)	2,125,511.75 (1,489,953.81–2,803,300.68)	59.76 (41.71–78.87)	0.54 (0.48–0.60)	0.85 (0.83–0.87)
SDI						
High SDI	414,696.48 (284,569.22–562,441.49)	121.26 (82.89–162.42)	442,030.26 (327,998.38–564,015.05)	136.33 (100.65–172.26)	0.07 (−0.03 to 0.18)	−0.02 (−0.17 to 0.13)
High-middle SDI	258,756.32 (176,987.63–343,213.34)	45.63 (31.17–60.87)	314,697.14 (216,659.74–418,076.31)	68.54 (46.48–91.50)	0.22 (0.18–0.25)	1.62 (1.55–1.69)
Middle SDI	481,044.41 (325,116.38–641,432.47)	46.46 (31.42–62.05)	789,535.23 (541,584.83–1,049,799.63)	77.15 (52.71–103.17)	0.64 (0.58–0.71)	1.78 (1.74–1.81)
Low-middle SDI	165,167.58 (109,661.33–221,466.63)	23.95 (15.91–31.96)	392,085.41 (260,976.06–527,327.97)	39.93 (26.61–53.81)	1.37 (1.27–1.49)	1.88 (1.83–1.93)
Low SDI	57,413.46 (36,760.68–78,232.89)	17.89 (11.66–24.02)	185,659.79 (120,988.00–253,847.89)	25.11 (16.57–33.98)	2.23 (2.09–2.38)	1.25 (1.21–1.29)
Regions						
Andean Latin America	23,567.43 (15,770.69–32,632.59)	93.87 (62.99–129.67)	40,400.74 (27,374.80–55,487.54)	129.25 (87.44–177.77)	0.71 (0.56–0.88)	1.11 (1.01–1.20)
Australasia	15,501.11 (11,070.70–20,379.13)	168.10 (120.91–222.32)	19,898.67 (13,292.62–26,417.01)	198.40 (131.79–263.05)	0.28 (0.04–0.48)	0.37 (0.25–0.48)
Caribbean	8,236.97 (5597.32–11,198.10)	39.70 (27.03–54.22)	11,196.08 (7529.72–15,205.13)	51.28 (34.37–69.96)	0.36 (0.28–0.46)	0.92 (0.84–0.99)
Central Asia	4741.59 (3042.62–6608.99)	12.16 (7.81–16.91)	7732.92 (5099.10–10,562.72)	18.00 (11.82–24.66)	0.63 (0.52–0.77)	1.28 (1.19–1.38)
Central Europe	3923.31 (2435.12–5555.08)	6.92 (4.30–9.84)	2983.59 (2015.28–4049.68)	8.62 (5.74–11.67)	−0.24 (−0.30 to −0.13)	0.73 (0.67–0.79)
Central Latin America	109,739.72 (72,552.04–152,105.39)	99.47 (66–137.49)	135,596.97 (91,275.47–182,692.45)	109.29 (72.81–147.93)	0.24 (0.19–0.29)	−0.17 (−0.31 to −0.03)
Central Sub-Saharan Africa	5106.08 (3170.29–7143.26)	14.99 (9.40–20.66)	19,766.07 (12,813.17–27,264.28)	22.85 (15.04–31.10)	2.87 (2.54–3.30)	1.33 (1.14–1.51)
East Asia	195,837.16 (129,426.78–264,360.64)	28.64 (18.85–38.87)	233,009.80 (159,243.18–310,679.21)	55.19 (37.04–74.38)	0.19 (0.10–0.29)	2.24 (2.02–2.46)
Eastern Europe	7384.03 (4805.21–10,158.51)	7.56 (4.89–10.43)	7023.63 (4673.06–9512.59)	10.26 (6.72–14.17)	−0.05 (−0.10 to 0.02)	1.05 (1.01–1.09)
Eastern Sub-Saharan Africa	21,781.74 (13,828.71–29,978.52)	17.69 (11.48–24.10)	63,827.35 (40,787.32–87,456.71)	22.94 (14.86–31.02)	1.93 (1.81–2.08)	0.90 (0.85–0.95)
High-income Asia Pacific	159,558.92 (107,237.28–225,168.40)	189.92 (127.49–263.94)	111,260.80 (74,602.31–154,461.62)	221.72 (150.41–306.85)	−0.30 (−0.34 to −0.25)	0.40 (0.32–0.48)
High-income North America	128,108.74 (85,651.80–173,345.59)	112.80 (74.99–153.83)	165,548.90 (130,928.93–203,599.87)	121.95 (96.40–149.86)	0.29 (0.05–0.65)	−0.82 (−1.22 to −0.42)
North Africa and Middle East	127,160.51 (85,003.98–172,459.17)	57.75 (38.86–77.75)	236,311.84 (158,280.10–322,447.39)	77.18 (51.59–105.45)	0.86 (0.78–0.94)	1.13 (1.06–1.20)
Oceania	1763.72 (1194.15–2367.45)	44.01 (29.85–59.17)	4635.16 (3094.39–6256.97)	63.28 (42.30–85.38)	1.63 (1.43–1.86)	1.01 (0.80–1.21)
South Asia	150,406.79 (100,769.25–201,388.96)	23.48 (15.86–31.29)	398,435.84 (267,815.43–532,993.57)	40.09 (26.96–53.64)	1.65 (1.50–1.82)	2.08 (1.93–2.23)
Southeast Asia	154,277.15 (100,925.66–208,964.64)	52.44 (34.48–70.78)	334,179.58 (226,904.14–448,458.32)	103.52 (70.49–138.85)	1.17 (1.02–1.37)	2.61 (2.51–2.71)
Southern Latin America	11,231.80 (7383.73–15,410.42)	42.42 (27.91–58.21)	20,675.42 (13,550.76–28,258.63)	71.35 (46.61–97.10)	0.84 (0.70–1.02)	1.81 (1.56–2.05)
Southern Sub-Saharan Africa	10,678.43 (6924.51–14,734.78)	30.33 (19.95–41.68)	16,360.68 (10,748.95–22,308.72)	39.13 (25.78–53.48)	0.53 (0.46–0.61)	0.86 (0.76–0.96)
Tropical Latin America	21,013.23 (13,910.36–28,452.57)	22.07 (14.73–29.71)	23,964.46 (16,224.83–31,992.16)	24.16 (16.11–32.47)	0.14 (0.09–0.20)	−0.24 (−0.40 to −0.07)
Western Europe	197,112.29 (133,859.72–265,911.60)	136.18 (91.91–185.21)	197,386.45 (134,522.21–266,089.31)	149.69 (101.44–203.13)	0.00 (−0.03 to 0.04)	0.22 (0.16–0.27)
Western Sub-Saharan Africa	20,793.87 (13,199.89–28,636.90)	17.14 (11.11–23.21)	75,316.82 (48,295.56–103,644.68)	24.45 (15.85–33.23)	2.62 (2.44–2.82)	0.97 (0.76–1.18)

ASIR, age-standardized incidence rate; EAPC, estimated annual percentage change; CI, confidence interval; UI, uncertainty interval; SDI, socio-demographic index.

**Table 2:** The DALYs and ASDR of PCOS at the regional level and its temporal trends from 1990 to 2019.

Characteristics	1990		2019		1990–2019	
	DALYs No. (95% UI)	ASDR per 100,000 No. (95% UI)	DALYs No. (95% UI)	ASDR per 100,000 No. (95% UI)	Change in DALYs No. (95% UI)	EAPC No. (95% CI)
Global	301,987.02 (127,370.28–606,719.05)	11.30 (4.78–22.67)	576,822.00 (246,221.54–1,161,158.56)	14.68 (6.28–29.45)	0.91 (0.83–0.99)	0.83 (0.78–0.87)
SDI						
High SDI	113,083.18 (48,472.41–231,333.99)	26.47 (11.39–54.41)	136,959.54 (60,261.65–276,121.82)	29.77 (13.12–59.72)	0.21 (0.12–0.32)	0.02 (−0.13 to 0.16)
High-middle SDI	62,264.06 (26,156.09–125,762.36)	10.39 (4.37–20.98)	107,048.95 (45,571.54–217,650.56)	15.07 (6.43–30.54)	0.72 (0.66–0.79)	1.30 (1.23–1.36)
Middle SDI	88,925.45 (37,318.46–180,282.61)	9.93 (4.18–20.18)	215,592.92 (92,249.83–437,657.80)	17.19 (7.34–34.90)	1.42 (1.32–1.55)	1.91 (1.84–1.98)
Low-middle SDI	28,399.10 (12,027.14–57,275.08)	5.28 (2.23–10.68)	86,016.85 (36,623.70–174,144.24)	9.20 (3.93–18.68)	2.03 (1.88–2.21)	2.05 (2.00–2.11)
Low SDI	9132.81 (3815.20–18,906.87)	3.88 (1.63–7.98)	30,816.60 (12,883.07–62,669.63)	5.63 (2.37–11.49)	2.37 (2.21–2.57)	1.34 (1.30–1.38)
Regions						
Andean Latin America	3798.53 (1648.30–7599.75)	19.71 (8.51–39.31)	9326.01 (4017.10–18,431.79)	27.98 (12.05–55.36)	1.46 (1.24–1.70)	1.28 (1.20–1.35)
Australasia	3685.32 (1561.73–7311.63)	34.30 (14.55–68.17)	5556.43 (2394.67–11,246.98)	40.44 (17.39–81.30)	0.51 (0.26–0.71)	0.38 (0.27–0.49)
Caribbean	1782.14 (748.16–3648.74)	9.51 (3.99–19.40)	2983.65 (1272.49–6059.21)	12.28 (5.23–24.99)	0.67 (0.57–0.81)	0.88 (0.82–0.95)
Central Asia	925.42 (379.86–1896.64)	2.71 (1.12–5.54)	2002.26 (845.91–4053.97)	4.07 (1.72–8.27)	1.16 (0.99–1.38)	1.35 (1.25–1.44)
Central Europe	939.55 (381.47–1926.14)	1.53 (0.62–3.15)	1002.81 (421.19–2029.11)	1.93 (0.81–3.94)	0.07 (−0.03 to 0.23)	0.80 (0.76–0.85)
Central Latin America	18,688.21 (8038.56–38,071.92)	21.96 (9.44–44.73)	33,434.11 (14,383.66–67,190.16)	24.68 (10.62–49.59)	0.79 (0.71–0.88)	−0.11 (−0.25 to 0.03)
Central Sub-Saharan Africa	803.19 (330.46–1672.42)	3.24 (1.34–6.70)	3183.32 (1329.87–6541.91)	5.05 (2.11–10.38)	2.96 (2.51–3.49)	1.42 (1.23–1.61)
East Asia	43,141.88 (18,028.46–88,103.56)	6.50 (2.72–13.21)	93,534.90 (39,875.60–188,707.23)	12.68 (5.37–25.69)	1.17 (1.02–1.34)	2.36 (2.14–2.59)
Eastern Europe	1861.09 (774.28–3776.25)	1.61 (0.67–3.27)	2258.59 (945.57–4572.90)	2.22 (0.91–4.53)	0.21 (0.14–0.31)	1.11 (1.06–1.15)
Eastern Sub-Saharan Africa	3284.18 (1373.53–6779.45)	3.82 (1.60–7.77)	10,343.00 (4304.13–21,355.31)	5.09 (2.15–10.49)	2.15 (1.99–2.32)	0.99 (0.94–1.04)
High-income Asia Pacific	37,775.62 (15,804.95–78,510.25)	40.88 (17.02–84.93)	36,294.87 (15,220.49–76,072.53)	45.20 (18.94–94.23)	−0.04 (−0.09 to 0.03)	0.27 (0.22–0.32)
High-income North America	37,486.44 (16,122.91–77,628.73)	24.97 (10.76–51.58)	45,346.81 (20,503.46–87,337.18)	27.05 (12.32–52.08)	0.21 (−0.01 to 0.53)	−0.76 (−1.15 to −0.38)
North Africa and Middle East	22,308.69 (9543.44–45,142.66)	13.74 (5.90–27.80)	59,834.76 (24,958.71–121,069.86)	18.71 (7.81–37.90)	1.68 (1.55–1.81)	1.26 (1.18–1.34)
Oceania	320.83 (135.41–643.87)	10.26 (4.33–20.48)	988.98 (426.48–2043.17)	14.65 (6.31–30.14)	2.08 (1.82–2.38)	0.99 (0.77–1.20)
South Asia	26,984.38 (11,200.13–54,435.17)	5.24 (2.19–10.56)	91701.66 (38,804.63–186,865.21)	9.56 (4.05–19.46)	2.40 (2.18–2.66)	2.35 (2.20–2.50)
Southeast Asia	29,232.35 (12,426.92–58,773.72)	12.07 (5.12–24.41)	85,843.31 (36,504.50–176,083.62)	23.78 (10.10–48.89)	1.94 (1.72–2.23)	2.58 (2.48–2.68)
Southern Latin America	2393.94 (1027.63–4951.80)	9.59 (4.11–19.83)	5577.71 (2336.60–11,645.77)	16.13 (6.74–33.64)	1.33 (1.13–1.57)	1.81 (1.56–2.06)
Southern Sub-Saharan Africa	1864.27 (774.96–3838.40)	6.87 (2.86–14.05)	3854.72 (1625.52–7808.76)	8.99 (3.80–18.22)	1.07 (0.97–1.19)	0.93 (0.85–1.01)
Tropical Latin America	3746.54 (1572.49–7535.46)	4.67 (1.97–9.42)	6368.82 (2702.19–12,892.41)	5.24 (2.23–10.65)	0.70 (0.62–0.80)	−0.07 (−0.21 to 0.07)
Western Europe	57,785.23 (24,718.93–114,977.07)	30.13 (12.91–59.93)	64,999.07 (27,584.75–130,922.40)	34.30 (14.53–69.27)	0.12 (0.08–0.17)	0.31 (0.22–0.40)
Western Sub-Saharan Africa	3179.25 (1308.93–6545.50)	3.67 (1.53–7.60)	12,386.19 (5168.62–25,696.21)	5.51 (2.32–11.33)	2.90 (2.68–3.14)	1.10 (0.88–1.33)

DALYs, disability-adjusted life years; ASDR, age-standardized disability-adjusted life years rate; EAPC, estimated annual percentage change; CI, confidence interval; UI, uncertainty interval; SDI, socio-demographic index.

## Data Availability

The data underlying this article are available in the Global Health Data Exchange at https://ghdx.healthdata.org/gbd-2019 (accessed on 5 June 2022).

## Supplementary Materials

The following supporting information can be downloaded at: https://www.mdpi.com/article/10.3390/healthcare11040562/s1, Table S1: The incidence and age-standardized incidence rate of PCOS at national level; Table S2: The disability-adjusted life years and age-standardized disability-adjusted life years rate of PCOS at national level.
