# Supplementary material for: Study of Burden in Polycystic Ovary Syndrome at Global, Regional, and National Levels from 1990 to 2019
Source: Healthcare (Basel). 2023 Feb 14;11(4):562. doi: 10.3390/healthcare11040562 (PMC9957370; doi:10.3390/healthcare11040562)
Supplement: Supplementary file 1 [file healthcare-11-00562-s001.zip › Table S1.pdf]

Table S1. The incidence and ASIR of PCOS at national level.

Characteristics	1990		2019		1990-2019
	Incident cases No. (95% UI)	ASIR per 100,000 No. (95% UI)	Incident cases No. (95% UI)	ASIR per 100,000 No. (95% UI)	EAPC No. (95%CI)
**Armenia**	191.52 (124.03-267.23)	11.34 (7.30-15.92)	185.33 (120.49-255.17)	18.39 (11.89-25.64)	1.75 (1.70-1.80)
**Azerbaijan**	460.11 (289.58-638.75)	11.49 (7.19-16.05)	761.48 (505.13-1054.27)	19.12 (12.57-26.54)	1.91 (1.77-2.05)
**Georgia**	361.58 (235.85-508.42)	14.52 (9.51-20.54)	318.82 (215.71-432.65)	27.68 (18.74-37.69)	2.49 (2.22-2.75)
**Kazakhstan**	1117.74 (726.03-1561.82)	12.92 (8.41-18.04)	1510.79 (974.73-2071.96)	19.67 (12.73-27.16)	1.38 (1.34-1.41)
**Kyrgyzstan**	302.62 (196.75-424.42)	11.79 (7.70-16.38)	480.50 (312.85-663.82)	14.86 (9.66-20.62)	0.54 (0.46-0.62)
**Mongolia**	157.41 (98.52-226.84)	11.34 (7.13-16.11)	241.15 (157.65-336.69)	17.02 (11.10-23.90)	1.39 (1.33-1.44)
**Tajikistan**	305.98 (192.00-428.41)	9.39 (5.95-13.06)	655.09 (428.87-920.08)	12.82 (8.39-17.99)	0.99 (0.90-1.07)
**Turkmenistan**	274.46 (172.23-390.69)	12.18 (7.67-17.40)	437.31 (284.20-609.67)	18.63 (12.09-26.04)	1.39 (1.35-1.43)
**Uzbekistan**	1570.18 (988.01-2266.50)	12.41 (7.87-17.76)	3142.44 (2095.59-4421.91)	18.47 (12.27-26.09)	1.26 (1.10-1.42)
**Albania**	104.58 (63.08-150.83)	5.46 (3.30-7.88)	73.97 (46.24-103.78)	7.39 (4.50-10.51)	1.00 (0.90-1.10)
**Bosnia and Herzegovina**	100.03 (59.64-144.63)	4.54 (2.67-6.64)	77.46 (49.55-108.87)	7.36 (4.59-10.45)	1.84 (1.61-2.08)
**Bulgaria**	233.32 (142.09-329.81)	6.48 (3.96-9.22)	162.11 (102.71-227.65)	8.40 (5.33-11.88)	0.89 (0.83-0.95)
**Croatia**	116.85 (70.46-166.95)	5.85 (3.51-8.43)	102.86 (65.49-144.68)	8.13 (5.11-11.62)	1.27 (1.14-1.39)
**Czechia**	287.18 (175.87-412.49)	5.99 (3.69-8.60)	247.32 (154.02-355.53)	8.02 (5.00-11.69)	0.91 (0.85-0.97)
**Hungary**	303.23 (184.63-436.34)	6.60 (4.03-9.51)	240.75 (151.38-341.02)	8.37 (5.24-12.05)	0.80 (0.74-0.87)
**Montenegro**	19.17 (11.82-28.36)	6.34 (3.87-9.42)	18.81 (11.46-26.66)	8.38 (5.04-11.97)	1.11 (1.01-1.21)
**North Macedonia**	52.3 (30.79-76.24)	5.26 (3.09-7.67)	53.25 (34.16-73.56)	7.54 (4.68-10.50)	1.32 (1.25-1.39)
**Poland**	1662.64 (1048.31-2338.53)	9.55 (6.03-13.43)	1113.42 (811.27-1448.12)	9.96 (7.19-13.02)	0.09 (0.03-0.15)
**Romania**	618.25 (371.05-893.05)	5.49 (3.30-7.93)	491.16 (298.56-699.38)	7.98 (4.83-11.43)	1.36 (1.32-1.40)

**Serbia**	229.66 (137.84-331.23)	5.49 (3.30-7.93)	222.52 (139.44-316.40)	7.56 (4.67-10.86)	1.26 (1.18-1.33)
**Slovakia**	144.44 (87.49-211.94)	5.65 (3.42-8.28)	130.66 (81.60-182.83)	8.03 (4.85-11.46)	1.17 (1.14-1.20)
**Slovenia**	51.67 (32.00-74.29)	6.09 (3.74-8.80)	49.31 (30.79-69.09)	8.75 (5.43-12.38)	1.29 (1.19-1.38)
**Belarus**	319.26 (202.31-448.83)	7.13 (4.44-10.00)	291.04 (188.33-400.59)	10.13 (6.49-14.13)	1.25 (1.17-1.33)
**Estonia**	49.51 (31.38-70.15)	7.64 (4.77-10.96)	45.34 (29.3-61.99)	11.45 (7.22-15.83)	1.60 (1.52-1.68)
**Latvia**	81.35 (50.42-115.43)	7.62 (4.69-10.86)	58.87 (37.92-81.37)	10.47 (6.65-14.71)	1.17 (1.09-1.25)
**Lithuania**	112.57 (71.48-157.54)	6.98 (4.37-9.89)	83.64 (54.18-114.63)	10.08 (6.40-14.11)	1.41 (1.31-1.51)
**Republic of Moldova**	127.38 (79.29-177.70)	6.04 (3.72-8.41)	113.94 (74.18-158.24)	9.23 (5.88-12.96)	1.56 (1.39-1.73)
**Russian Federation**	5063.71 (3258.57-6970.08)	7.75 (4.95-10.69)	5122.58 (3422.38-6988.73)	10.57 (6.92-14.65)	1.05 (1.02-1.08)
**Ukraine**	1630.25 (1070.97-2214.71)	7.29 (4.73-9.99)	1308.21 (857.32-1768.73)	9.27 (5.96-12.86)	0.85 (0.76-0.93)
**Australia**	11816.49 (8388.16-15593.00)	155.36 (111.24-204.31)	16077.26 (10598.83-21595.97)	192.80 (127.04-259.73)	0.54 (0.39-0.69)
**New Zealand**	3684.62 (2465.05-5062.92)	227.47 (150.91-310.37)	3821.41 (2614.75-5041.19)	226.24 (154.65-298.05)	-0.28 (-0.38--0.18)
**Brunei Darussalam**	137.03 (89.41-193.98)	96.50 (62.99-136.71)	314.53 (206.33-441.47)	161.09 (105.94-224.36)	1.86 (1.67-2.05)
**Japan**	137816.53 (92075.08-192978.42)	242.48 (163.46-336.47)	89573.34 (60126.15-124407.16)	264.33 (179.14-364.00)	0.29 (0.21-0.36)
**Republic of Korea**	20316.45 (13032.68-28869.90)	79.41 (51.27-111.02)	19236.57 (12856.41-27651.55)	130.78 (86.80-184.88)	1.27 (0.90-1.64)
**Singapore**	1288.91 (827.40-1827.93)	90.34 (57.61-126.44)	2136.36 (1400.39-3038.55)	156.49 (102.84-220.65)	1.94 (1.76-2.11)
**Canada**	5919.15 (3803.18-8261.97)	54.05 (34.82-75.20)	8307.27 (5406.47-11535.57)	70.67 (45.84-98.33)	0.89 (0.76-1.02)
**Greenland**	9.58 (6.24-13.11)	44.94 (28.83-61.89)	13.23 (8.63-18.17)	61.94 (40.35-84.87)	1.15 (1.05-1.26)
**United States of America**	122177.08 (81600.34-165727.27)	119.07 (79.06-162.48)	157225.76 (125037.43-193575.59)	126.81 (100.82-155.16)	-0.94 (-1.36--0.52)
**Argentina**	7330.31 (4800.40-10121.66)	41.61 (27.26-57.39)	13848.29 (9114.36-18857.79)	67.56 (44.42-92.61)	1.65 (1.47-1.84)
**Chile**	3253.89 (2136.70-4475.78)	44.31 (29.15-60.32)	5777.36 (3762.24-7977.35)	81.77 (52.96-112.10)	2.18 (1.83-2.53)
**Uruguay**	647.14 (417.20-897.67)	42.62 (27.48-59.07)	1048.73 (684.56-1455.25)	74.40 (48.12-102.37)	1.95 (1.74-2.16)
**Andorra**	22.64 (15.32-31.46)	107.64 (71.68-151.45)	32.53 (21.66-44.13)	138.89 (92.00-189.42)	0.82 (0.69-0.95)

**Austria**	3676.27 (2557.30-4962.57)	137.84 (95.46-186.31)	3644.16 (2462.02-5008.22)	149.33 (100.42-204.28)	0.05 (-0.06-0.15)
**Belgium**	4055.97 (2723.73-5619.71)	113.25 (75.19-156.12)	5019.16 (3405.95-6972.62)	139.02 (93.99-193.64)	0.58 (0.35-0.81)
**Cyprus**	288.98 (192.16-402.22)	82.24 (54.69-114.68)	474.03 (317.58-656.45)	126.78 (84.59-175.82)	1.60 (1.41-1.80)
**Denmark**	1930.59 (1281.54-2655.18)	99.53 (65.54-137.58)	2484.26 (1655.66-3407.84)	129.65 (86.03-178.14)	0.87 (0.74-1.01)
**Finland**	1774.85 (1187.78-2446.66)	99.45 (66.51-137.53)	2170.17 (1452.75-2967.14)	127.11 (84.99-173.58)	0.85 (0.79-0.91)
**France**	21095.99 (14316.82-28833.21)	90.95 (61.32-123.34)	27341.11 (18489.54-37239.36)	117.67 (79.18-160.58)	0.83 (0.77-0.88)
**Germany**	22539.04 (15006.27-31130.91)	93.22 (61.48-129.32)	25684.20 (17618.26-34799.91)	116.79 (78.69-157.62)	0.67 (0.62-0.73)
**Greece**	4714.15 (3194.87-6473.33)	108.23 (72.27-150.38)	4162.10 (2752.33-5900.38)	141.30 (93.08-200.96)	0.72 (0.49-0.95)
**Iceland**	131.55 (88.82-181.92)	111.68 (74.68-154.64)	180.47 (121.24-245.67)	144.24 (96.31-197.32)	0.94 (0.88-1.00)
**Ireland**	2023.55 (1361.47-2802.73)	103.19 (69.49-142.92)	2504.43 (1635.29-3527.98)	133.47 (87.12-188.06)	0.83 (0.71-0.95)
**Israel**	2453.86 (1647.22-3420.10)	90.4 (60.80-125.82)	5092.14 (3383.31-7151.62)	120.45 (80.07-169.29)	0.90 (0.79-1.02)
**Italy**	73673.08 (49975.75-100857.24)	344.51 (232.37-480.27)	50089.92 (33674.51-68381.22)	314.44 (211.23-431.29)	-0.64 (-0.78--0.50)
**Luxembourg**	128.63 (85.48-174.93)	105.34 (69.95-144.25)	262.74 (177.31-361.20)	141.36 (94.61-192.82)	0.98 (0.90-1.07)
**Malta**	157.55 (105.49-222.42)	97.78 (65.50-138.23)	159.43 (107.71-219.60)	139.23 (93.39-191.55)	1.16 (0.97-1.34)
**Monaco**	8.04 (5.44-11.12)	116.36 (77.50-161.00)	13.88 (9.34-19.10)	141.97 (94.70-196.03)	0.63 (0.55-0.72)
**Netherlands**	5175.27 (3452.76-7075.13)	91.63 (60.82-126.36)	6599.60 (4459.52-8945.65)	118.41 (79.17-161.34)	0.82 (0.76-0.89)
**Norway**	2087.04 (1412.92-2803.90)	128.30 (85.44-173.47)	2463.96 (1678.02-3306.10)	136.62 (92.07-183.23)	0.32 (0.29-0.35)
**Portugal**	4213.55 (2834.79-5732.87)	87.37 (58.91-118.77)	3840.42 (2558.82-5271.50)	124.12 (81.98-172.47)	0.94 (0.71-1.17)
**San Marino**	11.38 (7.72-15.74)	110.78 (74.90-154.01)	15.48 (10.31-21.21)	136.01 (89.97-187.92)	0.69 (0.58-0.80)
**Spain**	18458.18 (12322.22-25894.50)	101.23 (67.17-143.56)	17370.71 (11741.04-23986.85)	130.66 (88.08-180.43)	0.70 (0.53-0.86)
**Sweden**	2293.69 (1532.33-3104.81)	76.04 (50.63-103.15)	3111.89 (2103.45-4187.80)	95.11 (63.96-128.21)	0.44 (0.24-0.65)
**Switzerland**	2279.21 (1554.83-3173.00)	102.11 (69.12-142.91)	2851.57 (1905.75-3890.84)	118.95 (78.59-163.32)	0.51 (0.48-0.53)
**United Kingdom**	23755.57 (16260.66-31757.70)	113.46 (76.00-152.34)	31646.00 (21278.78-42508.78)	145.68 (97.10-196.09)	0.60 (0.43-0.77)

**Bolivia (Plurinational State of)**	3497.27 (2245.40-4906.40)	83.52 (54.53-116.58)	7075.77 (4563.77-9788.81)	114.71 (73.93-159.11)	1.06 (1.01-1.10)
**Ecuador**	7348.94 (4927.69-10164.05)	109.81 (73.87-151.61)	13624.73 (9392.49-18500.03)	147.06 (100.72-200.21)	1.01 (0.74-1.29)
**Peru**	12721.23 (8465.42-17617.31)	89.44 (59.55-123.65)	19700.24 (13031.15-27832.17)	124.51 (82.37-176.46)	1.12 (1.04-1.19)
**Antigua and Barbuda**	13.69 (9.08-18.66)	40.80 (27.02-55.73)	20.78 (13.67-28.55)	56.22 (36.36-78.31)	0.88 (0.79-0.97)
**Bahamas**	82.80 (54.08-115.27)	53.48 (34.72-74.67)	117.34 (75.99-163.59)	64.74 (41.59-90.23)	0.58 (0.48-0.68)
**Barbados**	66.39 (43.73-91.12)	52.47 (34.46-72.24)	67.93 (45.02-93.80)	63.00 (41.19-87.33)	0.53 (0.48-0.59)
**Belize**	52.53 (34.48-72.97)	40.93 (26.94-56.43)	157.84 (105.07-218.58)	61.90 (41.15-86.11)	1.19 (0.87-1.52)
**Bermuda**	14.63 (9.79-20.15)	66.21 (43.48-92.10)	13.36 (8.79-18.60)	73.40 (48.1-102.10)	0.24 (0.17-0.31)
**Cuba**	2428.16 (1653.71-3365.42)	45.10 (30.18-62.50)	2174.28 (1441.88-3004.15)	61.00 (40.35-85.45)	1.03 (0.98-1.08)
**Dominica**	17.97 (11.84-25.08)	40.84 (26.91-56.80)	17.97 (11.81-24.71)	57.48 (37.69-79.60)	1.01 (0.86-1.15)
**Dominican Republic**	1693.68 (1149.18-2315.55)	35.24 (23.87-48.05)	3172.42 (2110.79-4379.50)	58.11 (38.31-80.33)	1.74 (1.63-1.86)
**Grenada**	18.07 (11.84-25.16)	34.20 (22.52-47.51)	23.10 (15.26-31.61)	50.75 (33.54-70.22)	1.13 (0.96-1.30)
**Guyana**	185.03 (124.43-253.62)	35.65 (24.04-48.76)	211.12 (139.57-291.54)	52.59 (34.77-73.16)	1.28 (1.16-1.39)
**Haiti**	1059.47 (694.73-1491.19)	25.94 (17.14-36.07)	2462.61 (1644.92-3391.56)	33.36 (22.32-45.95)	0.91 (0.87-0.95)
**Jamaica**	596.28 (396.23-827.31)	39.20 (26.18-54.26)	732.11 (488.93-995.60)	54.89 (36.53-75.59)	1.08 (0.99-1.17)
**Puerto Rico**	1256.71 (839.70-1788.69)	64.73 (43.36-92.33)	1053.33 (707.53-1457.64)	84.91 (56.38-118.79)	0.93 (0.83-1.03)
**Saint Kitts and Nevis**	11.90 (7.85-16.40)	46.95 (31.08-64.67)	16.45 (10.94-22.62)	65.65 (43.32-91.10)	1.02 (0.90-1.14)
**Saint Lucia**	35.08 (22.66-49.10)	38.43 (24.90-53.68)	38.31 (25.36-52.95)	53.07 (34.96-73.69)	0.81 (0.62-1.00)
**Saint Vincent and the Grenadines**	25.91 (17.13-35.78)	34.57 (22.86-47.48)	27.56 (18.06-37.23)	54.44 (35.46-74.05)	1.59 (1.48-1.71)
**Suriname**	91.61 (60.76-127.74)	40.62 (26.98-56.75)	154.88 (101.34-214.21)	57.21 (37.28-79.12)	1.13 (1.10-1.16)
**Trinidad and Tobago**	276.38 (183.89-378.43)	40.54 (27.01-55.40)	324.82 (214.68-447.02)	61.82 (40.77-85.32)	1.53 (1.38-1.68)
**United States Virgin Islands**	36.30 (24.45-50.19)	62.20 (41.88-85.97)	30.59 (20.06-43.11)	80.94 (53.00-114.10)	0.85 (0.71-0.98)

**Colombia**	11907.75 (7845.69-16408.99)	58.95 (38.78-81.44)	17282.71 (11349.96-24059.76)	80.69 (52.90-113.11)	0.92 (0.85-0.99)
**Costa Rica**	1334.33 (884.84-1876.04)	73.62 (48.79-103.43)	2094.91 (1392.02-2906.39)	102.05 (68.00-142.10)	0.96 (0.88-1.05)
**El Salvador**	2189.67 (1425.23-3053.47)	59.23 (39.01-82.47)	2806.63 (1836.04-3855.91)	88.17 (57.66-121.77)	1.27 (1.09-1.45)
**Guatemala**	2645.35 (1727.70-3689.67)	49.81 (32.77-69.61)	7742.66 (5198.36-10801.36)	72.95 (48.57-101.79)	1.13 (0.99-1.27)
**Honduras**	1555.75 (1007.35-2155.76)	47.30 (30.89-64.92)	4544.02 (2922.46-6355.64)	74.99 (48.03-104.82)	1.57 (1.44-1.70)
**Mexico**	79250.35 (52336.18-109146.63)	132.89 (87.81-183.33)	84013.67 (56547.40-111925.69)	135.61 (91.27-181.21)	-0.61 (-0.79--0.43)
**Nicaragua**	1529.12 (1012.15-2190.78)	54.80 (36.60-78.02)	2943.43 (1967.87-4077.95)	81.29 (54.32-112.60)	1.26 (1.14-1.38)
**Panama**	726.90 (482.67-1024.67)	49.56 (32.95-70.04)	1692.54 (1094.69-2383.97)	83.66 (54.11-118.05)	1.57 (1.47-1.67)
**Venezuela (Bolivarian Republic of)**	8600.50 (5496.88-12220.48)	71.50 (45.72-101.48)	12476.40 (8129.16-17596.29)	94.43 (61.46-133.38)	0.79 (0.71-0.87)
**Brazil**	20680.82 (13687.63-27989.10)	22.30 (14.87-30.01)	23141.07 (15624.42-30908.73)	24.23 (16.20-32.60)	-0.28 (-0.45--0.11)
**Paraguay**	332.41 (210.29-474.54)	13.52 (8.59-19.19)	823.39 (555.06-1137.22)	22.19 (14.88-30.63)	1.90 (1.85-1.96)
**Afghanistan**	2435.03 (1619.47-3332.70)	31.31 (20.89-42.45)	11947.85 (8073.47-16278.16)	46.65 (31.56-63.38)	1.97 (1.62-2.32)
**Algeria**	9645.62 (6234.27-13321.86)	56.88 (37.12-78.22)	15931.70 (10376.32-22520.61)	85.64 (55.68-120.80)	1.62 (1.56-1.69)
**Bahrain**	194.62 (127.37-271.35)	81.65 (53.48-112.93)	448.33 (290.58-608.91)	94.63 (60.99-129.53)	0.48 (0.45-0.51)
**Egypt**	23587.74 (15338.77-32829.66)	69.85 (45.43-96.83)	49168.80 (32674.59-68384.67)	90.37 (60.13-125.33)	0.72 (0.65-0.80)
**Iran (Islamic Republic of)**	22820.99 (15266.63-30931.40)	59.94 (40.71-80.77)	28007.82 (19027.03-37686.95)	83.04 (56.19-110.94)	1.72 (1.29-2.15)
**Iraq**	8034.48 (5314.32-10909.23)	68.15 (45.26-92.53)	19121.08 (12617.37-26266.51)	77.10 (50.97-106.66)	0.64 (0.49-0.79)
**Jordan**	1647.87 (1081.07-2311.06)	62.70 (41.36-87.86)	5486.30 (3544.09-7778.00)	81.08 (52.51-114.78)	1.06 (0.98-1.13)
**Kuwait**	775.46 (512.12-1090.10)	89.22 (59.18-124.54)	1566.34 (1037.33-2185.51)	108.60 (71.67-151.36)	0.97 (0.88-1.06)
**Lebanon**	1219.60 (807.67-1699.20)	67.70 (44.82-94.42)	1859.55 (1237.72-2573.46)	89.70 (60.39-123.68)	1.03 (0.98-1.08)
**Libya**	2337.98 (1515.73-3194.53)	78.66 (51.22-107.46)	2805.02 (1826.90-3863.07)	87.50 (56.61-121.59)	0.54 (0.47-0.61)
**Morocco**	9391.74 (6270.09-12828.41)	57.44 (38.48-78.13)	13917.30 (9127.97-18930.93)	78.11 (51.21-106.76)	1.10 (1.08-1.12)

**Oman**	573.90 (369.53-802.54)	54.07 (35.59-74.47)	1435.75 (931.94-1977.75)	94.06 (60.75-130.00)	2.10 (2.01-2.19)
**Palestine**	770.82 (507.53-1079.02)	56.97 (37.91-79.54)	2280.84 (1477.49-3210.94)	72.86 (47.36-102.02)	0.86 (0.80-0.92)
**Qatar**	148.40 (96.92-210.35)	88.59 (58.31-124.38)	634.12 (409.99-903.24)	105.07 (68.01-147.92)	0.68 (0.61-0.75)
**Saudi Arabia**	6895.79 (4456.87-9503.67)	70.79 (46.09-97.34)	13508.90 (8808.95-18606.92)	103.04 (66.87-143.01)	1.43 (1.39-1.47)
**Sudan**	4696.42 (3128.30-6444.22)	36.06 (24.27-49.15)	17975.97 (11775.69-24965.11)	68.21 (44.80-94.71)	2.18 (2.00-2.37)
**Syrian Arab Republic**	5184.22 (3372.66-7235.17)	57.90 (38.27-79.88)	7741.48 (5126.70-10653.59)	78.40 (51.84-109.21)	1.15 (1.06-1.24)
**Tunisia**	2894.68 (1887.41-4008.00)	55.23 (36.17-76.19)	3799.44 (2517.08-5211.94)	80.92 (53.35-111.44)	1.46 (1.41-1.51)
**Turkey**	20070.93 (13335.96-27890.67)	53.40 (35.63-74.02)	26634.28 (17678.58-36724.22)	77.59 (51.34-107.42)	1.49 (1.43-1.54)
**United Arab Emirates**	522.36 (337.15-729.94)	71.19 (45.94-98.39)	2008.56 (1343.15-2811.76)	95.88 (63.96-133.10)	1.05 (0.90-1.19)
**Yemen**	3226.36 (2121.40-4372.56)	37.18 (24.79-49.53)	9792.34 (6528.08-13442.53)	47.12 (31.71-64.51)	1.18 (1.02-1.33)
**Bangladesh**	9201.00 (5685.04-12954.29)	13.22 (8.24-18.42)	20554.91 (13227.05-28291.97)	22.62 (14.58-31.12)	2.14 (1.96-2.31)
**Bhutan**	76.07 (48.60-106.79)	19.49 (12.58-27.02)	145.84 (97.72-203.55)	36.86 (24.58-51.63)	2.46 (2.34-2.58)
**India**	122844.91 (82744.01-163717.03)	25.06 (17.10-33.24)	332699.82 (225124.51-441702.27)	44.96 (30.31-60.01)	2.24 (2.06-2.42)
**Nepal**	1596.05 (998.35-2208.71)	13.51 (8.54-18.49)	4616.63 (3020.99-6282.00)	24.53 (16.09-33.34)	2.12 (2.04-2.20)
**Pakistan**	16688.76 (10639.70-23245.87)	24.52 (16.00-33.51)	40418.63 (26195.81-55180.93)	28.46 (18.66-38.59)	0.76 (0.62-0.89)
**China**	186317.06 (123142.63-252245.92)	28.11 (18.40-38.26)	223654.29 (152542.05-298625.60)	55.08 (36.80-74.26)	2.29 (2.06-2.53)
**Democratic People's Republic of Korea**	3408.14 (2256.11-4659.00)	30.40 (20.00-41.70)	3353.70 (2262.16-4573.09)	33.39 (22.22-46.45)	0.28 (0.17-0.39)
**Taiwan (Province of China)**	6111.95 (4081.12-8449.65)	56.74 (37.86-78.23)	6001.81 (4268.15-7966.37)	96.99 (68.08-129.71)	2.05 (1.97-2.13)
**American Samoa**	20.72 (14.05-28.69)	73.77 (50.07-102.22)	35.19 (23.22-48.18)	105.56 (68.85-145.31)	1.09 (0.85-1.34)
**Cook Islands**	8.78 (5.80-12.10)	76.42 (50.56-105.35)	9.70 (6.28-13.40)	117.70 (76.17-162.93)	1.35 (1.16-1.55)
**Fiji**	265.40 (176.68-360.27)	56.53 (37.68-76.27)	416.19 (271.28-575.55)	91.11 (59.57-125.74)	1.46 (1.27-1.65)
**Guam**	55.58 (36.62-77.75)	82.12 (53.99-114.76)	93.94 (62.26-131.29)	121.51 (80.57-169.47)	1.32 (1.21-1.43)

**Kiribati**	20.05 (13.39-27.57)	47.39 (31.66-65.20)	50.62 (33.84-68.30)	73.31 (49.24-98.75)	1.29 (1.01-1.58)
**Marshall Islands**	12.53 (8.10-17.36)	39.92 (26.06-54.32)	23.02 (15.35-31.63)	69.99 (46.87-96.59)	1.70 (1.49-1.92)
**Micronesia (Federated States of)**	38.40 (25.02-53.04)	53.38 (35.23-73.23)	49.37 (32.65-67.73)	79.31 (52.21-108.89)	1.15 (0.90-1.41)
**Nauru**	3.81 (2.51-5.22)	62.58 (41.54-85.95)	6.09 (4.03-8.49)	93.08 (61.61-129.90)	1.16 (1.06-1.27)
**Niue**	0.85 (0.56-1.19)	69.04 (45.80-94.96)	0.80 (0.53-1.09)	108.71 (71.79-148.69)	1.54 (1.32-1.76)
**Northern Mariana Islands**	18.82 (12.54-25.86)	78.37 (51.29-107.68)	19.96 (13.46-27.44)	103.66 (68.87-143.47)	0.80 (0.59-1.01)
**Palau**	6.43 (4.24-8.82)	72.69 (47.91-100.40)	7.31 (4.84-10.14)	108.80 (72.04-150.92)	1.25 (1.03-1.47)
**Papua New Guinea**	952.90 (631.90-1283.76)	38.00 (25.31-51.04)	3125.89 (2084.84-4241.57)	57.66 (38.47-78.21)	1.18 (0.97-1.39)
**Samoa**	73.33 (48.31-100.37)	64.68 (43.06-88.10)	124.82 (82.89-173.66)	89.84 (59.93-124.46)	0.98 (0.80-1.16)
**Solomon Islands**	91.19 (59.64-126.22)	39.5 (26.03-54.50)	256.21 (168.09-357.62)	64.23 (42.35-89.40)	1.46 (1.15-1.77)
**Tokelau**	0.54 (0.35-0.76)	56.68 (37.46-78.93)	0.65 (0.43-0.90)	96.35 (63.59-132.91)	1.72 (1.53-1.91)
**Tonga**	50.17 (33.56-68.30)	74.37 (50.20-100.55)	65.32 (43.05-91.95)	106.36 (70.19-149.73)	0.85 (0.57-1.13)
**Tuvalu**	2.47 (1.66-3.37)	52.13 (34.76-71.07)	5.45 (3.61-7.59)	85.99 (56.88-119.73)	1.48 (1.25-1.71)
**Vanuatu**	44.16 (29.28-60.53)	46.87 (31.33-64.03)	125.71 (84.21-171.50)	70.59 (47.39-96.53)	1.36 (1.29-1.42)
**Cambodia**	2545.63 (1620.83-3526.76)	39.14 (25.13-53.94)	6081.03 (3997.11-8307.64)	70.44 (46.16-96.23)	2.12 (2.10-2.15)
**Indonesia**	60831.96 (39647.14-82755.82)	51.33 (33.66-69.67)	135161.55 (92064.01-180625.53)	103.39 (70.31-138.70)	2.69 (2.59-2.79)
**Lao People's Democratic Republic**	1209.38 (780.60-1706.02)	44.55 (28.89-62.50)	3521.72 (2402.71-4765.12)	87.09 (59.31-117.40)	2.48 (2.43-2.53)
**Malaysia**	9441.73 (6321.31-12869.71)	88.44 (59.00-120.08)	23897.59 (16399.95-33208.10)	162.11 (110.00-226.77)	2.23 (2.05-2.40)
**Maldives**	69.12 (44.26-96.21)	47.96 (31.06-65.97)	210.79 (139.67-284.81)	123.64 (81.93-167.38)	4 (3.68-4.31)
**Mauritius**	542.84 (363.60-752.39)	86.79 (58.17-121.32)	719.23 (485.96-997.79)	145.58 (97.96-205.16)	1.89 (1.77-2.00)
**Myanmar**	10482.49 (6880.53-14105.05)	41.08 (27.06-55.17)	26102.45 (17411.77-35653.96)	91.29 (61.14-125.50)	3.22 (3.06-3.37)
**Philippines**	23263.76 (15176.37-31834.29)	56.86 (37.49-77.57)	61096.97 (41666.40-82238.28)	99.48 (67.78-133.36)	2.10 (2.02-2.19)

**Seychelles**	40.69 (27.21-55.78)	92.91 (62.37-126.88)	53.70 (36.53-73.17)	135.22 (91.02-185.12)	1.17 (1.10-1.24)
**Sri Lanka**	6914.43 (4476.36-9470.07)	67.89 (44.11-92.97)	12379.48 (8249.31-17218.36)	121.81 (81.36-169.83)	2.24 (1.97-2.51)
**Thailand**	21871.80 (14432.21-30123.84)	64.00 (42.55-87.90)	31480.67 (21322.42-43233.99)	135.54 (91.27-187.90)	2.63 (2.43-2.82)
**Timor-Leste**	176.35 (112.99-239.08)	42.10 (27.00-57.06)	661.00 (436.74-908.40)	73.08 (48.59-100.17)	2.22 (2.12-2.33)
**Viet Nam**	16681.89 (10813.79-22820.68)	38.75 (25.18-53.01)	32375.58 (21345.00-44431.97)	83.22 (54.57-114.25)	3.20 (2.99-3.40)
**Angola**	852.76 (543.21-1197.59)	13.54 (8.72-18.69)	4956.01 (3198.60-6885.87)	24.85 (16.39-34.05)	1.92 (1.74-2.09)
**Central African Republic**	299.59 (189.60-419.10)	17.88 (11.41-24.83)	683.64 (436.74-953.43)	19.46 (12.63-26.82)	0.01 (-0.14-0.16)
**Congo**	319.56 (200.16-441.17)	19.46 (12.47-26.74)	873.11 (558.10-1203.87)	27.20 (17.46-37.28)	1.01 (0.85-1.16)
**Democratic Republic of the Congo**	3457.36 (2114.82-4856.65)	14.68 (9.09-20.40)	12538.24 (8068.18-17346.45)	21.69 (14.3-29.69)	1.25 (1.04-1.45)
**Equatorial Guinea**	40.78 (25.32-57.09)	15.71 (9.91-21.76)	346.24 (225.51-478.48)	38.94 (25.70-53.61)	3.40 (2.96-3.85)
**Gabon**	136.02 (84.23-190.45)	21.94 (13.93-30.52)	368.84 (239.98-511.26)	33.89 (22.08-46.77)	1.35 (1.19-1.50)
**Burundi**	499.88 (317.65-695.69)	14.84 (9.63-20.41)	1270.64 (786.13-1780.50)	16.11 (10.14-22.35)	0.16 (0.10-0.23)
**Comoros**	69.08 (42.81-97.60)	22.22 (14.16-30.92)	112.72 (73.10-156.63)	26.71 (17.42-37.12)	0.35 (0.15-0.55)
**Djibouti**	50.10 (30.64-71.73)	17.23 (10.62-24.34)	174.87 (112.92-242.30)	30.21 (19.58-41.93)	2.07 (1.93-2.20)
**Eritrea**	241.43 (150.66-335.31)	12.80 (8.08-17.82)	868.09 (543.44-1191.13)	19.78 (12.49-26.98)	1.39 (1.16-1.63)
**Ethiopia**	4117.48 (2670.01-5616.62)	13.33 (8.87-17.97)	14568.83 (9345.94-20022.09)	20.29 (13.14-27.50)	1.58 (1.50-1.66)
**Kenya**	3212.91 (2031.76-4425.82)	20.41 (13.28-27.71)	8264.85 (5281.29-11303.90)	24.53 (15.91-33.30)	0.48 (0.38-0.57)
**Madagascar**	1238.71 (787.12-1734.99)	16.14 (10.46-22.46)	3284.09 (2085.31-4559.72)	18.90 (12.07-25.98)	0.55 (0.50-0.61)
**Malawi**	1373.87 (878.47-1916.49)	22.19 (14.48-30.83)	3577.66 (2258.88-5075.13)	25.78 (16.46-35.97)	0.61 (0.47-0.74)
**Mozambique**	1361.61 (838.81-1899.98)	15.76 (9.92-21.84)	5008.61 (3238.20-7062.16)	24.34 (15.87-34.04)	1.49 (1.39-1.59)
**Rwanda**	918.13 (578.83-1291.53)	19.83 (12.64-27.62)	2145.67 (1390.95-2985.61)	25.32 (16.51-35.14)	0.97 (0.90-1.03)
**Somalia**	717.73 (441.32-1021.11)	16.06 (10.14-22.26)	2508.32 (1598.65-3498.99)	19.08 (12.37-26.23)	0.70 (0.65-0.74)

**South Sudan**	663.24 (414.46-925.30)	17.81 (11.32-24.53)	1280.85 (814.76-1792.11)	20.08 (12.91-27.83)	0.20 (0.06-0.33)
**Uganda**	2094.4 (1297.77-2912.42)	18.25 (11.63-25.09)	6745.68 (4290.36-9272.91)	23.34 (15.05-31.80)	0.83 (0.80-0.86)
**United Republic of Tanzania**	3777.34 (2323.93-5287.48)	21.37 (13.54-29.51)	10051.02 (6415.18-14070.76)	26.58 (17.15-36.78)	0.70 (0.59-0.80)
**Zambia**	1429.84 (902.66-1994.77)	24.84 (15.89-34.39)	3914.41 (2474.33-5475.67)	31.09 (20.01-43.28)	0.58 (0.52-0.64)
**Botswana**	230.98 (148.62-330.25)	24.31 (15.87-34.15)	501.97 (332.90-691.79)	39.38 (26.07-54.33)	2.09 (1.93-2.25)
**Eswatini**	189.10 (122.27-261.01)	32.40 (21.32-44.25)	294.91 (194.85-412.68)	41.50 (27.60-58.01)	0.52 (0.23-0.82)
**Lesotho**	262.38 (166.26-369.41)	21.44 (13.68-30.01)	424.48 (279.55-582.90)	33.35 (22.06-45.78)	1.49 (1.36-1.62)
**Namibia**	224.21 (142.98-316.73)	23.26 (15.08-32.57)	463.99 (309.23-641.01)	32.01 (21.39-44.26)	1.06 (0.91-1.21)
**South Africa**	7786.27 (5041.14-10705.37)	32.29 (21.06-44.28)	11757.20 (7765.54-15933.74)	43.41 (28.50-59.02)	1.08 (1.01-1.14)
**Zimbabwe**	1985.47 (1274.89-2801.34)	26.67 (17.56-37.17)	2918.12 (1909.58-4056.27)	29.11 (19.09-40.35)	-0.07 (-0.29-0.15)
**Benin**	451.42 (283.74-634.39)	15.67 (9.99-21.97)	2457.81 (1569.76-3444.28)	29.36 (18.91-40.90)	2.11 (1.82-2.39)
**Burkina Faso**	882.86 (551.04-1242.48)	14.76 (9.37-20.41)	3543.10 (2283.60-4920.23)	24.07 (15.65-33.18)	1.44 (1.21-1.66)
**Cabo Verde**	38.39 (23.99-53.61)	17.08 (10.88-23.65)	85.40 (54.04-117.37)	28.86 (18.27-39.81)	1.62 (1.38-1.86)
**Cameroon**	1553.67 (980.91-2170.11)	23.69 (15.32-32.68)	6288.94 (3926.45-8787.08)	32.07 (20.44-44.90)	0.80 (0.68-0.92)
**Chad**	420.33 (265.27-588.77)	11.44 (7.36-15.73)	1907.64 (1201.04-2662.93)	17.39 (11.04-23.80)	1.02 (0.76-1.29)
**Côte d'Ivoire**	1175.41 (734.95-1635.73)	15.79 (10.02-21.69)	4132.31 (2605.18-5701.95)	26.45 (16.83-36.45)	1.62 (1.30-1.94)
**Gambia**	99.51 (62.40-138.46)	15.87 (10.19-21.90)	376.69 (244.97-527.65)	24.05 (15.83-33.55)	1.13 (0.94-1.32)
**Ghana**	1534.69 (951.29-2135.70)	16.50 (10.39-22.74)	4847.32 (3082.45-6731.84)	24.88 (15.88-34.53)	1.10 (0.81-1.39)
**Guinea**	504.92 (315.01-707.78)	14.34 (9.10-19.95)	1825.92 (1135.11-2532.22)	21.81 (13.78-30.19)	1.23 (1.08-1.39)
**Guinea-Bissau**	94.03 (59.74-133.30)	14.40 (9.20-20.03)	277.02 (176.14-383.21)	21.97 (14.15-30.22)	1.22 (0.92-1.52)
**Liberia**	207.55 (129.79-289.75)	16.40 (10.55-22.62)	785.74 (501.98-1091.25)	24.61 (15.92-33.92)	1.53 (1.44-1.63)
**Mali**	618.84 (386.63-873.91)	12.26 (7.80-17.08)	2894.65 (1861.01-4075.28)	19.75 (13.07-27.42)	1.39 (1.17-1.61)
**Mauritania**	264.21 (164.18-366.26)	20.59 (13.07-28.50)	813.36 (514.91-1114.29)	29.20 (18.78-39.89)	0.83 (0.60-1.06)

**Niger**	643.45 (399.97-885.06)	12.88 (8.19-17.52)	2729.87 (1693.93-3802.35)	17.43 (11.10-23.76)	0.99 (0.83-1.15)
**Nigeria**	10698.85 (6863.18-14705.44)	18.43 (12.04-25.01)	37538.24 (23968.72-51413.08)	24.92 (16.27-33.77)	0.77 (0.56-0.98)
**Sao Tome and Principe**	14.06 (8.82-19.93)	17.42 (11.16-24.21)	35.89 (22.97-49.67)	26.08 (16.91-35.61)	1.17 (0.93-1.41)
**Senegal**	931.31 (576.39-1321.16)	18.56 (11.70-25.72)	2293.59 (1466.03-3176.15)	23.72 (15.31-32.68)	0.56 (0.40-0.71)
**Sierra Leone**	308.91 (194.96-435.68)	14.13 (8.94-19.72)	1369.66 (884.91-1913.51)	24.63 (16.13-34.22)	1.70 (1.55-1.85)
**Togo**	350.76 (217.11-491.52)	14.84 (9.35-20.59)	1112.61 (708.63-1545.45)	23.10 (14.97-32.03)	1.20 (1.00-1.40)

ASIR, age-standardized incidence rate; EAPC, estimated annual percentage change; CI, confidence interval; UI, uncertainty interval.
