# Supplementary material for: Study of Burden in Polycystic Ovary Syndrome at Global, Regional, and National Levels from 1990 to 2019
Source: Healthcare (Basel). 2023 Feb 14;11(4):562. doi: 10.3390/healthcare11040562 (PMC9957370; doi:10.3390/healthcare11040562)
Supplement: Supplementary file 1 [file healthcare-11-00562-s001.zip › Table S2.pdf]

Table S2. The DALYs and ASDR of PCOS at national level.

Characteristics	1990		2019		1990-2019
	DALYs No. (95% UI)	ASDR per 100,000 No. (95% UI)	DALYs No. (95% UI)	ASDR per 100,000 No. (95% UI)	EAPC No. (95%CI)
**Armenia**	43.88 (17.58-89.81)	2.48 (0.99-5.11)	61.80 (25.27-126.26)	4.08 (1.67-8.31)	1.76 (1.72-1.81)
**Azerbaijan**	97.77 (39.21-203.21)	2.55 (1.02-5.26)	241.00 (99.19-493.46)	4.32 (1.77-8.92)	2.02 (1.87-2.16)
**Georgia**	90.88 (37.24-187.75)	3.24 (1.33-6.71)	102.30 (42.56-208.13)	6.24 (2.59-12.85)	2.52 (2.26-2.79)
**Kazakhstan**	242.84 (98.38-497.54)	2.89 (1.17-5.90)	417.70 (174.86-843.70)	4.41 (1.84-8.89)	1.41 (1.37-1.44)
**Kyrgyzstan**	55.36 (22.39-112.36)	2.59 (1.05-5.30)	112.13 (45.81-227.65)	3.30 (1.34-6.69)	0.51 (0.42-0.61)
**Mongolia**	25.65 (10.67-53.18)	2.49 (1.03-5.12)	69.40 (28.10-145.10)	3.80 (1.54-7.91)	1.46 (1.40-1.51)
**Tajikistan**	50.15 (20.86-103.73)	2.05 (0.87-4.18)	141.07 (58.51-288.53)	2.84 (1.18-5.82)	1.05 (0.95-1.14)
**Turkmenistan**	48.26 (19.53-101.39)	2.69 (1.09-5.60)	104.80 (42.54-213.37)	4.18 (1.70-8.50)	1.45 (1.41-1.50)
**Uzbekistan**	270.64 (113.67-547.35)	2.72 (1.15-5.56)	752.07 (312.54-1537.26)	4.13 (1.72-8.45)	1.27 (1.10-1.44)
**Albania**	20.31 (7.84-43.26)	1.21 (0.47-2.57)	21.23 (8.72-45.69)	1.65 (0.67-3.54)	1.01 (0.90-1.11)
**Bosnia and Herzegovina**	23.59 (9.22-47.99)	0.99 (0.38-2.01)	24.69 (9.72-50.17)	1.62 (0.64-3.32)	1.88 (1.64-2.12)
**Bulgaria**	59.73 (24.76-123.59)	1.43 (0.59-2.97)	54.78 (21.57-112.07)	1.85 (0.72-3.78)	0.90 (0.84-0.95)
**Croatia**	31.42 (12.43-65.96)	1.29 (0.51-2.71)	33.26 (13.40-69.16)	1.80 (0.72-3.77)	1.28 (1.16-1.41)
**Czechia**	68.64 (27.52-145.53)	1.33 (0.53-2.80)	82.98 (32.39-168.76)	1.77 (0.69-3.66)	0.89 (0.83-0.95)
**Hungary**	74.04 (29.87-157.44)	1.45 (0.58-3.08)	81.60 (32.45-171.92)	1.86 (0.74-3.94)	0.84 (0.77-0.91)
**Montenegro**	4.43 (1.77-9.06)	1.40 (0.56-2.86)	5.46 (2.23-11.51)	1.86 (0.76-3.90)	1.12 (1.02-1.23)
**North Macedonia**	11.92 (4.58-24.75)	1.15 (0.44-2.38)	17.49 (7.13-35.64)	1.66 (0.68-3.37)	1.34 (1.27-1.42)
**Poland**	407.00 (166.32-833.70)	2.13 (0.87-4.35)	402.46 (172.60-816.76)	2.26 (0.97-4.61)	0.15 (0.12-0.19)
**Romania**	137.11 (54.07-278.70)	1.20 (0.47-2.44)	150.63 (61.71-307.55)	1.77 (0.71-3.67)	1.41 (1.37-1.46)

**Serbia**	55.09 (21.38-115.42)	1.20 (0.46-2.50)	65.85 (26.17-135.84)	1.67 (0.66-3.44)	1.28 (1.21-1.35)
**Slovakia**	33.13 (12.99-70.00)	1.24 (0.48-2.62)	45.95 (17.80-92.69)	1.78 (0.68-3.62)	1.19 (1.16-1.23)
**Slovenia**	13.14 (5.39-27.05)	1.31 (0.54-2.69)	16.45 (6.77-33.66)	1.89 (0.77-3.88)	1.32 (1.22-1.42)
**Belarus**	81.28 (32.11-167.73)	1.56 (0.61-3.23)	99.92 (41.30-204.02)	2.25 (0.92-4.56)	1.32 (1.23-1.41)
**Estonia**	13.09 (5.27-26.82)	1.68 (0.67-3.46)	14.73 (6.18-30.55)	2.57 (1.06-5.35)	1.70 (1.62-1.78)
**Latvia**	22.18 (8.86-44.74)	1.68 (0.67-3.40)	19.17 (7.88-39.07)	2.32 (0.95-4.72)	1.21 (1.13-1.29)
**Lithuania**	28.37 (11.40-57.88)	1.51 (0.60-3.10)	27.28 (11.21-56.58)	2.22 (0.91-4.62)	1.47 (1.37-1.58)
**Republic of Moldova**	29.93 (11.60-60.36)	1.30 (0.51-2.63)	37.84 (15.62-77.04)	2.05 (0.84-4.25)	1.65 (1.48-1.81)
**Russian Federation**	1274.04 (528.97-2569.85)	1.64 (0.68-3.35)	1623.95 (672.62-3306.49)	2.28 (0.94-4.70)	1.12 (1.09-1.15)
**Ukraine**	412.22 (171.14-834.92)	1.56 (0.64-3.15)	435.70 (175.89-889.82)	2.00 (0.81-4.12)	0.89 (0.80-0.98)
**Australia**	2846.83 (1210.79-5617.49)	31.84 (13.51-62.78)	4588.17 (1978.10-9298.53)	39.25 (16.98-78.91)	0.52 (0.38-0.67)
**New Zealand**	838.48 (361.82-1706.84)	46.38 (20.06-94.28)	968.26 (415.24-1998.55)	47.17 (20.30-97.54)	-0.15 (-0.22--0.07)
**Brunei Darussalam**	27.68 (11.48-57.46)	20.30 (8.34-42.10)	83.86 (36.02-174.42)	33.44 (14.37-69.48)	1.81 (1.62-2.00)
**Japan**	33106.00 (13926.42-68620.71)	51.38 (21.52-106.23)	28279.15 (11811.56-59132.75)	54.81 (23.02-115.14)	0.20 (0.14-0.26)
**Republic of Korea**	4280.25 (1772.46-9001.31)	16.79 (6.98-35.49)	6937.74 (2905.16-14689.35)	27.47 (11.54-57.95)	1.27 (0.92-1.62)
**Singapore**	361.69 (148.89-760.47)	19.07 (7.84-40.02)	994.11 (415.75-2106.63)	32.56 (13.61-68.62)	1.89 (1.72-2.06)
**Canada**	1808.39 (751.78-3743.32)	12.14 (5.07-24.93)	2603.92 (1099.44-5428.80)	15.89 (6.74-32.74)	0.89 (0.76-1.02)
**Greenland**	3.02 (1.26-6.16)	9.95 (4.11-20.32)	3.63 (1.53-7.47)	13.81 (5.84-28.34)	1.18 (1.07-1.28)
**United States of America**	35674.17 (15299.40-74019.30)	26.38 (11.37-54.61)	42738.54 (19395.20-82347.03)	28.25 (12.91-54.29)	-0.87 (-1.29--0.46)
**Argentina**	1520.05 (646.44-3111.68)	9.39 (3.99-19.26)	3584.72 (1518.39-7488.89)	15.20 (6.43-31.73)	1.64 (1.45-1.84)
**Chile**	728.07 (309.52-1515.82)	10.00 (4.25-20.87)	1711.09 (723.95-3552.96)	18.38 (7.82-38.05)	2.17 (1.82-2.52)
**Uruguay**	145.72 (60.81-296.99)	9.62 (4.01-19.63)	281.62 (118.83-583.15)	16.72 (7.07-34.58)	1.94 (1.72-2.16)
**Andorra**	7.48 (3.16-15.16)	24.95 (10.52-50.66)	12.56 (5.23-24.94)	31.73 (13.21-62.90)	0.78 (0.65-0.91)

**Austria**	1267.38 (544.18-2579.94)	32.00 (13.75-65.24)	1370.90 (581.99-2751.64)	34.41 (14.63-69.23)	0.00 (-0.10-0.11)
**Belgium**	1290.03 (536.92-2639.49)	26.33 (10.95-53.84)	1606.06 (672.68-3191.93)	32.14 (13.47-63.85)	0.57 (0.34-0.80)
**Cyprus**	76.15 (31.38-154.04)	19.16 (7.93-38.66)	202.86 (84.68-405.53)	29.02 (12.18-57.90)	1.54 (1.34-1.73)
**Denmark**	570.35 (237.32-1143.33)	21.85 (9.05-43.76)	734.37 (300.67-1440.30)	28.90 (11.79-56.59)	0.95 (0.79-1.12)
**Finland**	562.32 (238.07-1122.38)	22.11 (9.32-43.90)	664.75 (277.51-1351.84)	28.98 (12.11-59.10)	0.88 (0.80-0.96)
**France**	6284.50 (2571.86-12420.59)	21.68 (8.90-42.90)	8013.40 (3309.03-16032.35)	28.04 (11.54-56.15)	0.83 (0.77-0.89)
**Germany**	8212.21 (3384.17-16444.13)	20.85 (8.61-41.75)	9173.15 (3880.32-18642.18)	26.20 (11.07-52.77)	0.69 (0.62-0.76)
**Greece**	1270.98 (532.61-2573.24)	25.07 (10.56-50.76)	1439.52 (598.47-2887.00)	32.21 (13.43-64.32)	0.67 (0.44-0.90)
**Iceland**	33.94 (14.40-69.62)	25.99 (11.06-53.38)	52.62 (21.89-106.66)	32.98 (13.71-66.96)	0.89 (0.83-0.95)
**Ireland**	423.52 (175.48-851.25)	23.90 (9.91-48.08)	717.65 (301.36-1453.44)	30.49 (12.84-62.39)	0.80 (0.68-0.92)
**Israel**	505.60 (212.28-1015.51)	20.58 (8.64-41.20)	1184.59 (496.13-2395.77)	27.21 (11.42-55.03)	0.86 (0.75-0.98)
**Italy**	20492.56 (8819.05-40425.01)	71.87 (30.97-141.81)	17339.38 (7488.63-34682.30)	69.7 (30.20-139.85)	-0.36 (-0.48--0.24)
**Luxembourg**	47.71 (19.52-94.13)	24.36 (10.03-48.11)	97.29 (40.31-196.89)	32.17 (13.28-65.33)	0.95 (0.87-1.03)
**Malta**	42.99 (17.95-86.43)	22.69 (9.47-45.75)	60.80 (25.18-122.33)	31.76 (13.10-64.29)	1.10 (0.92-1.28)
**Monaco**	3.73 (1.56-7.46)	26.90 (11.14-55.01)	4.70 (1.95-9.40)	32.35 (13.48-64.59)	0.60 (0.51-0.68)
**Netherlands**	1689.84 (698.77-3417.10)	21.33 (8.78-43.05)	2034.79 (847.69-4112.45)	27.22 (11.28-55.54)	0.79 (0.72-0.85)
**Norway**	620.11 (259.10-1258.48)	29.46 (12.36-59.77)	755.80 (327.03-1545.47)	31.13 (13.42-63.77)	0.30 (0.26-0.33)
**Portugal**	1030.77 (432.39-2078.08)	20.27 (8.50-40.88)	1352.29 (566.59-2747.26)	28.45 (11.78-58.31)	0.90 (0.67-1.13)
**San Marino**	3.04 (1.29-6.23)	25.65 (10.83-52.53)	4.96 (2.03-9.84)	31.09 (12.70-61.68)	0.65 (0.54-0.76)
**Spain**	4248.35 (1792.54-8431.82)	21.97 (9.26-43.59)	5929.45 (2461.53-11887.79)	28.98 (12.11-58.25)	0.81 (0.64-0.99)
**Sweden**	732.77 (314.35-1490.34)	17.91 (7.64-36.63)	969.79 (410.47-1991.44)	22.05 (9.26-45.01)	0.39 (0.19-0.59)
**Switzerland**	829.22 (347.60-1683.37)	23.53 (9.85-47.71)	1070.31 (444.23-2185.50)	27.18 (11.28-55.61)	0.49 (0.47-0.51)
**United Kingdom**	7491.69 (3188.13-15513.72)	26.21 (11.21-54.30)	10150.41 (4310.30-20770.83)	33.26 (14.18-67.76)	0.58 (0.41-0.74)

**Bolivia (Plurinational State of)**	551.92 (237.32-1109.45)	17.78 (7.59-35.50)	1531.26 (652.47-3040.88)	24.93 (10.63-49.41)	1.15 (1.07-1.23)
**Ecuador**	1216.75 (508.42-2479.31)	23.68 (9.93-48.01)	2967.36 (1274.18-5909.30)	31.92 (13.74-63.48)	1.14 (0.92-1.37)
**Peru**	2029.86 (863.55-4032.59)	18.41 (7.82-36.64)	4827.38 (2041.85-9609.70)	26.97 (11.41-53.71)	1.38 (1.34-1.42)
**Antigua and Barbuda**	3.09 (1.28-6.40)	9.44 (3.91-19.46)	6.39 (2.65-13.07)	13.09 (5.44-26.67)	0.90 (0.81-0.98)
**Bahamas**	18.21 (7.61-37.66)	12.41 (5.20-25.71)	31.65 (12.98-63.98)	15.05 (6.17-30.50)	0.60 (0.50-0.69)
**Barbados**	16.78 (6.94-34.07)	12.18 (5.05-24.72)	20.95 (8.92-42.20)	14.67 (6.28-29.46)	0.54 (0.49-0.60)
**Belize**	8.23 (3.41-16.50)	9.49 (3.93-19.10)	32.99 (13.48-66.63)	14.30 (5.85-28.97)	1.18 (0.86-1.49)
**Bermuda**	5.24 (2.20-10.67)	15.41 (6.47-31.4)	4.75 (2.00-9.53)	17.09 (7.26-34.52)	0.25 (0.19-0.32)
**Cuba**	636.58 (269.41-1320.58)	10.43 (4.40-21.58)	738.90 (306.54-1500.34)	14.19 (5.98-29.01)	1.06 (1.01-1.11)
**Dominica**	3.27 (1.37-6.86)	9.44 (3.96-19.70)	4.46 (1.92-9.07)	13.36 (5.75-27.11)	1.03 (0.88-1.17)
**Dominican Republic**	311.97 (128.93-629.58)	8.14 (3.35-16.39)	766.43 (317.99-1551.99)	13.35 (5.54-27.02)	1.80 (1.70-1.90)
**Grenada**	3.12 (1.33-6.52)	7.86 (3.31-16.23)	6.19 (2.60-12.71)	11.77 (4.97-24.12)	1.15 (0.98-1.33)
**Guyana**	33.77 (13.54-67.45)	8.14 (3.26-16.26)	51.30 (21.02-104.40)	12.16 (5.00-24.76)	1.34 (1.21-1.47)
**Haiti**	183.70 (77.27-382.65)	5.88 (2.47-12.16)	528.73 (221.32-1077.35)	7.63 (3.19-15.48)	0.95 (0.90-0.99)
**Jamaica**	110.47 (45.63-226.53)	9.11 (3.79-18.43)	198.70 (81.93-407.21)	12.80 (5.28-26.17)	1.10 (1.01-1.19)
**Puerto Rico**	289.95 (119.37-587.61)	15.06 (6.20-30.53)	321.42 (132.31-642.94)	19.75 (8.10-39.29)	0.93 (0.84-1.03)
**Saint Kitts and Nevis**	2.21 (0.92-4.52)	10.86 (4.55-22.28)	4.76 (2.01-9.61)	15.27 (6.44-30.65)	1.04 (0.92-1.16)
**Saint Lucia**	6.16 (2.58-12.57)	8.86 (3.73-17.93)	11.48 (4.80-23.59)	12.30 (5.12-25.43)	0.83 (0.64-1.02)
**Saint Vincent and the Grenadines**	4.28 (1.79-8.67)	7.94 (3.35-16.10)	7.13 (2.96-14.26)	12.63 (5.26-25.28)	1.63 (1.51-1.74)
**Suriname**	18.33 (7.70-37.91)	9.35 (3.92-19.28)	38.79 (16.26-80.59)	13.25 (5.54-27.62)	1.14 (1.10-1.17)
**Trinidad and Tobago**	59.07 (24.04-121.36)	9.48 (3.86-19.35)	98.96 (40.06-201.34)	14.49 (5.86-29.30)	1.55 (1.40-1.70)
**United States Virgin Islands**	8.33 (3.41-17.17)	14.50 (5.95-29.89)	8.60 (3.55-17.31)	18.87 (7.85-38.10)	0.85 (0.72-0.98)

**Colombia**	2355.16 (1012.21-4764.21)	13.37 (5.76-27.13)	4665.92 (1973.09-9342.41)	18.41 (7.81-36.94)	0.98 (0.93-1.04)
**Costa Rica**	266.52 (111.31-546.92)	16.85 (7.03-34.35)	603.05 (246.75-1241.26)	23.30 (9.55-47.99)	0.97 (0.88-1.05)
**El Salvador**	356.04 (153.28-713.49)	13.36 (5.76-26.74)	697.13 (291.59-1424.01)	19.72 (8.27-40.17)	1.31 (1.16-1.45)
**Guatemala**	406.25 (167.70-823.47)	11.08 (4.59-22.53)	1638.66 (687.89-3311.94)	16.36 (6.90-32.96)	1.17 (1.03-1.30)
**Honduras**	229.81 (97.56-466.00)	10.62 (4.49-21.48)	922.85 (388.86-1873.45)	16.86 (7.10-34.16)	1.54 (1.42-1.67)
**Mexico**	13105.87 (5660.85-26824.14)	29.53 (12.66-60.08)	20684.78 (8941.44-41511.75)	30.38 (13.15-61.06)	-0.61 (-0.79--0.42)
**Nicaragua**	226.99 (96.10-470.39)	12.31 (5.19-25.42)	652.39 (276.23-1311.16)	18.28 (7.73-36.82)	1.27 (1.14-1.40)
**Panama**	138.36 (57.42-279.88)	11.17 (4.62-22.50)	395.50 (163.51-802.53)	18.88 (7.81-38.29)	1.57 (1.47-1.68)
**Venezuela (Bolivarian Republic of)**	1603.21 (684.33-3258.78)	16.27 (6.94-33.03)	3173.84 (1337.80-6410.09)	21.48 (9.08-43.54)	0.79 (0.71-0.87)
**Brazil**	3688.59 (1547.15-7412.85)	4.71 (1.98-9.48)	6183.18 (2625.56-12512.76)	5.25 (2.23-10.64)	-0.11 (-0.26-0.03)
**Paraguay**	57.96 (24.28-119.41)	3.07 (1.28-6.32)	185.65 (76.77-380.39)	5.05 (2.10-10.37)	1.93 (1.86-1.99)
**Afghanistan**	368.44 (155.17-746.07)	7.17 (3.03-14.48)	1950.76 (800.52-3924.48)	10.79 (4.52-21.63)	2.02 (1.65-2.38)
**Algeria**	1592.13 (650.42-3297.78)	13.26 (5.41-27.56)	4450.89 (1848.46-9057.60)	19.90 (8.20-40.38)	1.62 (1.54-1.69)
**Bahrain**	45.18 (18.63-92.44)	19.09 (7.92-39.21)	144.33 (59.24-299.51)	22.20 (9.06-45.60)	0.49 (0.46-0.52)
**Egypt**	4500.55 (1927.64-9189.29)	16.81 (7.20-34.37)	10824.45 (4444.82-22023.76)	21.33 (8.75-43.39)	0.66 (0.59-0.73)
**Iran (Islamic Republic of)**	3889.18 (1634.26-7901.30)	14.51 (6.15-29.25)	9318.73 (3926.77-18992.89)	20.05 (8.49-40.71)	1.72 (1.31-2.14)
**Iraq**	1246.90 (508.31-2523.38)	15.86 (6.60-31.99)	4099.68 (1741.81-8360.24)	18.03 (7.69-36.74)	0.66 (0.51-0.81)
**Jordan**	252.70 (105.68-511.35)	14.56 (6.05-29.44)	1117.25 (479.90-2281.01)	19.18 (8.19-39.41)	1.06 (0.97-1.16)
**Kuwait**	181.06 (74.36-364.38)	20.93 (8.64-41.84)	685.30 (287.55-1395.70)	25.40 (10.69-51.15)	0.96 (0.87-1.05)
**Lebanon**	244.90 (102.79-500.94)	15.77 (6.64-32.19)	564.19 (234.71-1141.66)	20.90 (8.78-42.04)	1.05 (1.01-1.10)
**Libya**	333.23 (138.97-668.38)	18.25 (7.61-36.80)	803.67 (328.18-1655.86)	20.47 (8.35-42.27)	0.58 (0.50-0.65)
**Morocco**	1736.34 (738.09-3536.44)	13.47 (5.75-27.37)	3544.54 (1480.91-7243.20)	18.41 (7.69-37.52)	1.17 (1.13-1.22)

**Oman**	88.86 (37.37-179.59)	12.67 (5.29-25.51)	425.54 (182.26-878.52)	22.04 (9.44-45.18)	2.10 (2.01-2.20)
**Palestine**	120.40 (48.85-244.58)	13.30 (5.51-26.94)	434.65 (178.13-892.97)	17.05 (7.00-35.26)	0.86 (0.80-0.92)
**Qatar**	33.31 (13.56-68.61)	20.76 (8.49-42.49)	225.72 (94.46-454.00)	24.62 (10.36-49.05)	0.68 (0.61-0.75)
**Saudi Arabia**	1127.00 (459.63-2326.28)	16.63 (6.84-34.07)	4709.71 (1972.70-9612.28)	24.18 (10.09-49.25)	1.43 (1.39-1.47)
**Sudan**	786.98 (330.42-1647.10)	8.23 (3.44-17.04)	3369.73 (1412.97-7043.34)	15.66 (6.54-32.66)	2.20 (2.01-2.40)
**Syrian Arab Republic**	758.83 (312.85-1532.33)	13.27 (5.45-27.10)	1450.68 (613.46-2964.27)	18.13 (7.65-37.12)	1.18 (1.08-1.28)
**Tunisia**	555.86 (237.83-1138.31)	13.24 (5.66-26.84)	1165.29 (500.99-2399.56)	19.04 (8.26-39.10)	1.43 (1.38-1.48)
**Turkey**	3827.55 (1635.27-7788.07)	12.71 (5.44-25.85)	8006.15 (3375.05-16345.95)	18.10 (7.63-37.12)	1.39 (1.33-1.44)
**United Arab Emirates**	117.03 (47.39-235.26)	16.67 (6.72-33.23)	747.52 (313.53-1512.30)	22.47 (9.43-45.67)	1.05 (0.90-1.19)
**Yemen**	487.27 (205.09-992.13)	8.60 (3.63-17.22)	1735.17 (729.31-3519.74)	10.91 (4.61-21.96)	1.17 (1.01-1.34)
**Bangladesh**	1444.22 (597.57-2930.84)	2.89 (1.19-5.90)	4488.53 (1842.40-9157.20)	4.98 (2.05-10.15)	2.16 (1.99-2.33)
**Bhutan**	12.09 (4.93-25.09)	4.29 (1.77-8.78)	34.22 (14.33-70.86)	8.29 (3.47-17.16)	2.55 (2.42-2.68)
**India**	22733.03 (9476.06-45654.93)	5.57 (2.33-11.23)	79398.96 (33651.92-161428.50)	10.73 (4.55-21.84)	2.55 (2.38-2.73)
**Nepal**	269.41 (112.86-560.12)	2.94 (1.23-6.10)	974.00 (404.52-1988.74)	5.50 (2.29-11.18)	2.17 (2.11-2.22)
**Pakistan**	2525.62 (1042.63-5093.53)	5.33 (2.23-10.60)	6805.96 (2855.05-13718.26)	6.15 (2.57-12.46)	0.55 (0.36-0.75)
**China**	40963.07 (17168.61-83649.33)	6.38 (2.68-12.98)	90036.27 (38360.94-181836.22)	12.63 (5.34-25.64)	2.41 (2.17-2.65)
**Democratic People's Republic of Korea**	763.15 (316.86-1554.03)	6.77 (2.84-13.81)	992.42 (416.49-1986.93)	7.43 (3.11-14.81)	0.27 (0.16-0.38)
**Taiwan (Province of China)**	1415.66 (594.25-2862.45)	12.73 (5.34-25.70)	2506.21 (1068.35-5092.27)	21.76 (9.16-44.36)	2.05 (1.96-2.13)
**American Samoa**	4.21 (1.74-8.53)	17.2 (7.13-34.53)	6.78 (2.85-13.97)	24.42 (10.25-50.23)	1.08 (0.84-1.32)
**Cook Islands**	1.66 (0.70-3.45)	17.87 (7.55-37.25)	2.39 (0.97-4.94)	27.35 (11.08-56.70)	1.33 (1.14-1.52)
**Fiji**	52.23 (21.84-104.47)	13.16 (5.55-26.24)	97.08 (40.25-199.27)	21.13 (8.76-43.36)	1.46 (1.27-1.64)
**Guam**	13.50 (5.63-27.49)	19.17 (7.99-39.06)	22.07 (9.36-45.38)	28.23 (12.01-58.20)	1.30 (1.19-1.40)

**Kiribati**	4.16 (1.70-8.18)	10.95 (4.51-21.61)	10.71 (4.55-21.54)	16.96 (7.20-34.08)	1.31 (1.02-1.59)
**Marshall Islands**	1.87 (0.77-3.77)	9.26 (3.83-18.51)	4.83 (2.04-9.85)	16.24 (6.88-33.06)	1.71 (1.51-1.92)
**Micronesia (Federated States of)**	5.87 (2.43-11.99)	12.41 (5.21-25.29)	9.70 (4.10-19.96)	18.40 (7.72-37.94)	1.15 (0.90-1.41)
**Nauru**	0.72 (0.30-1.50)	14.59 (6.07-30.44)	1.22 (0.50-2.49)	21.61 (8.87-43.97)	1.15 (1.05-1.26)
**Niue**	0.16 (0.07-0.33)	16.13 (6.65-32.47)	0.19 (0.08-0.39)	25.25 (10.35-51.59)	1.52 (1.31-1.74)
**Northern Mariana Islands**	5.08 (2.11-10.54)	18.30 (7.59-38.15)	4.74 (1.95-9.72)	24.08 (9.97-49.73)	0.78 (0.58-0.99)
**Palau**	1.41 (0.61-2.88)	16.98 (7.34-34.77)	1.97 (0.82-4.03)	25.26 (10.49-51.74)	1.23 (1.01-1.46)
**Papua New Guinea**	170.58 (71.87-340.83)	8.71 (3.67-17.43)	671.46 (286.49-1371.12)	13.32 (5.68-27.17)	1.21 (0.99-1.42)
**Samoa**	11.36 (4.64-23.77)	15.10 (6.12-31.91)	21.44 (9.19-43.45)	20.90 (8.90-42.63)	0.97 (0.79-1.15)
**Solomon Islands**	13.90 (5.89-27.72)	9.13 (3.93-18.25)	49.36 (20.23-99.75)	14.89 (6.11-30.07)	1.47 (1.16-1.78)
**Tokelau**	0.09 (0.04-0.19)	13.21 (5.45-26.90)	0.14 (0.06-0.29)	22.40 (9.13-45.57)	1.72 (1.53-1.90)
**Tonga**	7.96 (3.30-15.94)	17.38 (7.23-35.40)	12.53 (5.41-25.52)	24.69 (10.58-50.02)	0.83 (0.56-1.11)
**Tuvalu**	0.59 (0.25-1.21)	12.14 (5.09-24.69)	1.14 (0.48-2.32)	19.95 (8.41-40.60)	1.48 (1.26-1.71)
**Vanuatu**	7.75 (3.23-15.53)	10.85 (4.56-21.50)	24.52 (10.25-50.22)	16.37 (6.82-33.54)	1.35 (1.29-1.42)
**Cambodia**	438.59 (184.33-886.81)	8.71 (3.69-17.58)	1405.40 (588.39-2871.61)	15.78 (6.60-32.22)	2.15 (2.12-2.18)
**Indonesia**	11403.46 (4832.13-23035.62)	11.77 (5.00-23.73)	33613.48 (14439.19-68811.04)	23.74 (10.19-48.57)	2.62 (2.54-2.71)
**Lao People's Democratic Republic**	197.53 (85.37-394.50)	10.08 (4.34-20.15)	770.89 (329.09-1557.86)	19.76 (8.44-40.18)	2.49 (2.44-2.55)
**Malaysia**	1791.93 (769.69-3645.12)	19.77 (8.55-40.26)	6046.80 (2513.33-12611.76)	36.23 (15.05-75.53)	2.23 (2.05-2.41)
**Maldives**	10.61 (4.53-21.55)	10.93 (4.68-21.95)	64.36 (26.66-131.34)	28.28 (11.70-57.79)	4.03 (3.71-4.34)
**Mauritius**	119.98 (50.54-243.56)	19.71 (8.29-39.97)	212.69 (91.60-438.28)	32.90 (14.19-67.70)	1.87 (1.76-1.98)
**Myanmar**	1911.05 (808.79-3819.00)	9.27 (3.88-18.66)	6221.95 (2593.57-12791.97)	20.66 (8.61-42.55)	3.24 (3.08-3.39)
**Philippines**	4003.60 (1698.78-8174.41)	12.84 (5.48-26.14)	13121.21 (5647.90-26465.58)	22.80 (9.80-45.90)	2.35 (2.22-2.47)

**Seychelles**	7.76 (3.26-15.37)	21.13 (8.90-41.87)	14.98 (6.34-30.42)	30.63 (12.95-62.49)	1.15 (1.08-1.22)
**Sri Lanka**	1453.35 (617.49-3037.61)	15.67 (6.68-32.66)	3099.91 (1275.79-6266.34)	27.60 (11.32-55.89)	2.21 (1.95-2.47)
**Thailand**	4832.53 (2064.33-10027.31)	14.97 (6.38-30.97)	11222.23 (4722.04-22752.48)	31.22 (13.12-62.49)	2.60 (2.41-2.79)
**Timor-Leste**	36.00 (15.47-72.22)	9.56 (4.12-19.06)	109.90 (47.18-226.13)	16.69 (7.17-34.31)	2.24 (2.13-2.35)
**Viet Nam**	2987.12 (1240.73-5998.81)	8.64 (3.60-17.37)	9827.04 (4042.67-20260.42)	18.59 (7.62-38.35)	3.18 (2.98-3.38)
**Angola**	134.94 (55.47-274.79)	2.92 (1.20-5.94)	792.52 (325.46-1614.62)	5.52 (2.29-11.28)	2.03 (1.85-2.21)
**Central African Republic**	51.86 (21.33-105.97)	4.00 (1.67-8.23)	114.71 (46.47-233.66)	4.32 (1.76-8.78)	0.00 (-0.15-0.15)
**Congo**	47.91 (19.61-97.72)	4.23 (1.74-8.65)	163.31 (67.64-338.19)	5.96 (2.49-12.34)	1.00 (0.81-1.19)
**Democratic Republic of the Congo**	540.23 (219.85-1145.73)	3.15 (1.29-6.62)	1978.73 (824.30-4002.52)	4.75 (1.98-9.67)	1.32 (1.11-1.53)
**Equatorial Guinea**	6.70 (2.85-13.79)	3.39 (1.44-7.05)	59.80 (24.70-123.90)	8.77 (3.60-18.09)	3.57 (3.10-4.04)
**Gabon**	21.55 (8.82-44.04)	4.87 (2.01-9.87)	74.25 (30.70-150.35)	7.61 (3.14-15.46)	1.35 (1.18-1.52)
**Burundi**	81.20 (32.96-167.39)	3.21 (1.33-6.65)	195.22 (79.18-398.57)	3.49 (1.44-7.18)	0.17 (0.11-0.24)
**Comoros**	10.87 (4.44-22.18)	5.11 (2.12-10.46)	22.95 (9.44-47.20)	6.16 (2.54-12.67)	0.45 (0.26-0.64)
**Djibouti**	8.04 (3.35-16.49)	3.82 (1.57-7.77)	42.02 (17.47-85.27)	6.80 (2.83-13.89)	2.15 (2.00-2.30)
**Eritrea**	38.19 (15.99-78.17)	2.79 (1.16-5.61)	149.39 (61.41-303.05)	4.40 (1.81-8.98)	1.47 (1.24-1.70)
**Ethiopia**	636.42 (258.73-1298.73)	2.81 (1.17-5.69)	2336.14 (954.97-4781.35)	4.47 (1.88-9.15)	1.74 (1.65-1.83)
**Kenya**	449.51 (187.33-923.8)	4.38 (1.83-8.96)	1438.06 (609.91-2949.95)	5.50 (2.33-11.24)	0.67 (0.57-0.77)
**Madagascar**	191.82 (79.01-396.59)	3.56 (1.48-7.38)	563.12 (230.49-1146.83)	4.21 (1.74-8.52)	0.63 (0.57-0.69)
**Malawi**	219.54 (88.95-458.63)	4.93 (2.05-10.27)	531.71 (217.57-1093.79)	5.68 (2.33-11.74)	0.55 (0.42-0.67)
**Mozambique**	212.70 (84.97-434.74)	3.45 (1.39-7.03)	774.66 (318.62-1556.42)	5.41 (2.21-11.11)	1.54 (1.43-1.65)
**Rwanda**	138.80 (57.37-286.14)	4.26 (1.75-8.72)	367.43 (154.54-745.30)	5.51 (2.32-11.15)	0.93 (0.81-1.04)
**Somalia**	108.21 (44.35-221.08)	3.51 (1.45-7.20)	383.81 (156.77-792.06)	4.19 (1.73-8.61)	0.74 (0.69-0.79)

**South Sudan**	101.09 (41.57-209.59)	3.92 (1.61-8.19)	195.21 (80.65-392.33)	4.42 (1.83-8.89)	0.21 (0.07-0.35)
**Uganda**	310.71 (125.51-645.23)	4.01 (1.64-8.36)	1029.31 (423.69-2119.15)	5.20 (2.13-10.68)	0.85 (0.80-0.89)
**United Republic of Tanzania**	571.57 (236.31-1199.40)	4.72 (1.96-9.93)	1678.20 (701.08-3473.31)	5.92 (2.44-12.23)	0.74 (0.62-0.85)
**Zambia**	203.08 (81.31-414.05)	5.47 (2.21-11.13)	627.52 (256.00-1293.13)	6.78 (2.77-13.99)	0.49 (0.40-0.58)
**Botswana**	34.54 (14.07-71.37)	5.29 (2.12-11.00)	115.83 (48.67-237.64)	8.65 (3.64-17.79)	2.12 (1.96-2.29)
**Eswatini**	28.75 (11.98-59.60)	7.26 (3.06-15.11)	58.23 (24.72-120.59)	9.22 (3.91-19.10)	0.48 (0.19-0.77)
**Lesotho**	41.00 (16.45-84.33)	4.78 (1.97-9.79)	85.31 (35.69-174.99)	7.49 (3.15-15.28)	1.52 (1.40-1.65)
**Namibia**	34.89 (14.26-71.26)	5.12 (2.10-10.36)	92.09 (38.21-185.59)	7.09 (2.95-14.19)	0.99 (0.82-1.15)
**South Africa**	1440.92 (600.10-2930.42)	7.31 (3.06-14.99)	2994.45 (1267.39-6022.12)	9.81 (4.15-19.78)	1.08 (1.03-1.14)
**Zimbabwe**	284.18 (116.12-585.03)	5.84 (2.37-11.94)	508.81 (211.74-1017.25)	6.41 (2.66-12.73)	-0.17 (-0.41-0.07)
**Benin**	73.63 (31.27-149.20)	3.34 (1.42-6.76)	397.43 (163.40-813.76)	6.49 (2.67-13.46)	2.16 (1.88-2.45)
**Burkina Faso**	136.33 (55.70-277.38)	3.19 (1.32-6.48)	578.38 (233.61-1222.87)	5.28 (2.13-11.20)	1.49 (1.26-1.71)
**Cabo Verde**	6.00 (2.46-12.39)	3.75 (1.54-7.78)	19.33 (8.11-40.58)	6.48 (2.74-13.53)	1.68 (1.43-1.93)
**Cameroon**	255.27 (103.62-524.94)	5.30 (2.15-10.93)	1079.09 (446.91-2267.72)	7.28 (3.03-15.43)	0.81 (0.68-0.93)
**Chad**	63.44 (26.01-128.08)	2.40 (0.99-4.92)	269.82 (111.90-555.49)	3.78 (1.57-7.82)	1.07 (0.78-1.37)
**Côte d'Ivoire**	186.82 (74.81-385.28)	3.40 (1.40-7.06)	751.48 (304.85-1537.81)	5.87 (2.43-11.95)	1.71 (1.37-2.05)
**Gambia**	15.69 (6.44-32.03)	3.45 (1.43-7.04)	62.07 (25.47-128.35)	5.33 (2.20-10.95)	1.21 (1.01-1.41)
**Ghana**	251.30 (102.15-520.91)	3.54 (1.43-7.35)	960.48 (391.46-1954.43)	5.51 (2.26-11.27)	1.15 (0.87-1.44)
**Guinea**	86.96 (35.67-178.39)	3.07 (1.27-6.31)	300.03 (123.54-620.24)	4.80 (1.97-9.81)	1.26 (1.09-1.43)
**Guinea-Bissau**	14.67 (6.15-30.67)	3.09 (1.30-6.46)	48.36 (20.00-97.78)	4.83 (2.02-9.80)	1.30 (0.98-1.62)
**Liberia**	31.20 (12.58-63.96)	3.56 (1.45-7.34)	135.14 (57.56-280.46)	5.41 (2.30-11.13)	1.59 (1.49-1.68)
**Mali**	100.59 (41.55-205.60)	2.61 (1.09-5.34)	437.29 (179.71-900.18)	4.35 (1.79-8.93)	1.52 (1.28-1.77)
**Mauritania**	43.13 (17.50-88.97)	4.55 (1.84-9.36)	135.36 (55.41-279.31)	6.62 (2.71-13.78)	0.91 (0.68-1.15)

**Niger**	98.08 (41.41-199.40)	2.82 (1.19-5.74)	373.01 (153.20-775.44)	3.81 (1.58-7.88)	0.93 (0.77-1.10)
**Nigeria**	1564.85 (648.21-3217.98)	3.92 (1.63-8.10)	5994.78 (2518.26-12441.18)	5.63 (2.39-11.57)	0.91 (0.68-1.14)
**Sao Tome and Principe**	1.92 (0.80-3.92)	3.71 (1.55-7.58)	6.05 (2.45-12.56)	5.66 (2.34-11.78)	1.23 (0.98-1.47)
**Senegal**	142.36 (58.55-292.37)	4.10 (1.70-8.47)	397.09 (168.13-819.63)	5.30 (2.26-10.75)	0.59 (0.43-0.75)
**Sierra Leone**	52.31 (21.77-106.69)	3.01 (1.25-6.13)	231.43 (93.89-478.59)	5.42 (2.21-11.24)	1.76 (1.60-1.93)
**Togo**	54.58 (23.05-114.25)	3.19 (1.34-6.69)	209.42 (84.29-435.61)	5.08 (2.05-10.48)	1.33 (1.13-1.53)

DALY, disability-adjusted life years; ASDR, age-standardized disability-adjusted life years rate; EAPC, estimated annual percentage change; CI, confidence interval; UI, uncertainty interval; SDI, socio-demographic index.
